# Deoxyshikonin inhibits growth and induces apoptosis of hypertrophic scar-derived fibroblasts by downregulating FBXO expression through autophagy

**DOI:** 10.1038/s41598-026-49808-1

**Published:** 2026-04-28

**Authors:** Meirong Yan, Renzhang Liang, Lifeng Guan, Qiyun Luo, Xiaoyan Zhang, Hua Tian, Xuejun Wu

**Affiliations:** 1 Department of Burns, Plastic and Aesthetic Surgery, General Hospital of Ningxia Medical University, Yinchuan, 750004 Ningxia China; 2 Department of Pediatric Surgery, Peking University First Hospital Ningxia Women and Children’s Hospital (Ningxia Hui Autonomous Region Maternal and Child Health Hospital), Yinchuan, 750002 Ningxia China

**Keywords:** Hypertrophic scar, Deoxyshikonin, FBXO2, Apoptosis, Autophagy, ERK1/2, Cell biology, Diseases, Drug discovery, Molecular biology

## Abstract

Hypertrophic scar (HS) is a fibro-proliferative disorder of cutaneous wound healing, which seriously impacts the patient’s quality of life. Deoxyshikonin (DSK) as a natural shikonin derivative possesses various pharmacological effects, however, whether DSK is involved in anti-fibrotic function for HS remain unclear. This study aimed to investigate the effect of DSK on HS-derived fibroblasts (HSFs) growth and reveal its possible mechanism. HSFs were isolated from human HS tissues. The viability and proliferation of HSFs under DSK treatment were evaluated by CCK-8 and EdU assays. The evaluation of the apoptotic ratio was performed using flow cytometry. The effects of DSK on FBXO2 expressions, apoptosis-associated proteins, autophagy-related proteins and the molecules involved in Mitogen-activated protein kinase (MAPK) family were evaluated by western blot and immunofluorescence staining. mRFP-GFP-LC3 overexpression adenovirus was employed to detect autophagosome formation. Co-immunoprecipitation (Co-IP) assays were performed to examined the interaction between p62 and FBXO2. DSK was found to inhibit the proliferation and induce apoptosis of HSFs. Furthermore, we found that DSK suppressed the expression of FBXO2, and FBXO2 overexpression alleviated the effect of DSK on proliferation and apoptosis of HSFs. DSK possessed capability to induce autophagy by which elevating FBXO2 degradation by p62 interaction. Inhibiting autophagy using Atg5 shRNA increased FBXO2 expression and decreased apoptotic protein expression under DSK stimulation. Mechanistically, DSK-induced autophagy mediated the down-regulation of FBXO2 by activating extracellular regulated kinase 1/2 (ERK1/2) signaling. In conclusion, our finding demonstrated that DSK inhibited proliferation and induced apoptosis of HSFs by autophagy-mediated degradation of FBXO2 via activating ERK1/2 signaling. DSK emerges as a promising strategy to enhance outcome of hypertrophic scar.

**Supplementary Information:**

The online version contains supplementary material available at 10.1038/s41598-026-49808-1.

## Introduction

Hypertrophic scar (HS) is a fibro-proliferative disorder of cutaneous wound healing, which usually develops after severe burn injury or skin trauma^1^. It has been reported that the overall incidence of HS after skin trauma is 40%−70%^2^. HS has many adverse consequences including disfiguring, pain, itching, contracture, and motion restriction, seriously impacting patient’s quality of life (QOL)^3^. The traditional treatments for HS include surgery, silicone gel treatment, laser therapy, light therapy, drug therapy and radiotherapy^4^. However, the current treatments cannot efficiently attain scar-less healing or reverse fibrosis^5^. Thus, new therapeutic strategies are needed to manage HS effectively.

Previously, Traditional Chinese medicine (TCM), with advantages such as rich resource, relatively low cost and few side effects, showed excellent potential for scar treatments^6,7^. Shikonin is a naturally occurring naphthoquinone extracted from the oriental traditional medical herb *Lithospermum erythrorhizon*, which has been extensively used for the treatment of dermatitis, burns, and wounds^8^. Deoxyshikonin (DSK) is a natural shikonin derivative. It has been demonstrated that DSK possess various pharmacological effects including antibacterial, wound healing, and anticancer effects^9^. Furthermore, published studies have revealed that DSK exhibits its pharmacological effects partially by the process of autophagy^10,11^. Autophagy is an important homeostatic pathway that facilitates the degradation and recycling of cellular material^12^. Abnormalities of autophagy can promote the progression of many human diseases including HS^13^. It has been reported that autophagy is closely related to the maintenance, activity, differentiation, and life-death of skin fibroblasts during wound repair, which results in pathological scars^6^. Recently, autophagy activation has been suggested as a promising approach for HS treatment^14,15^. Given the link between DSK and autophagy, as well as the involvement of autophagy in scar formation, it is rational to hypothesize that DSK possesses the ability to promote HS repair. However, the potential of DSK as an effective drug for HS, along with its associated mechanisms, remains uncertain.

F-box only protein 2 (FBXO2), a well-known E3 ligase belonging to F-box family, plays an indispensable role in the ubiquitin-proteasome system and participates in various pathological processes^13,16^. Previously, the relationship between E3 ubiquitin ligases and autophagy has been established^17^. The F-box E3 ubiquitin ligases have been reported to mediate the degradation of target proteins through selective autophagy pathway^18^. Due to above findings, we hypothesized that DSK could inhibit the growth of HSFs by regulating FBXO2 and autophagy. To validate this hypothesis, we conducted this study, with the aim to investigate the effect of DSK on HSFs growth and reveal its possible mechanism.

## Methods

### Cell culture

HS samples were collected from three different patients of the Department of Plastic and Reconstructive Surgery at Ningxia Medical University General Hospital, with informed consent obtained from all participants prior to adipose tissue donation. All procedures involving human tissues and cells were approved by the Human Research Ethics Committee of Ningxia Medical University. All methods were performed in accordance with the ARRIVE guidelines and regulations of the Declaration of Helsinki. HS-derived fibroblasts (HSFs) were isolated according to a previously described protocol^19^. Briefly, the subcutaneous fat tissue was removed, and HSs samples were cut into small pieces. Then, samples were digested with 0.05% trypsin (Gibco, Grand Island, USA) and 0.1% collagenase type I (Gibco) at 37 °C for 2 h separately. Normal skin fibroblasts (NSFs) were isolated from adjacent healthy skin tissues of the same patients using the same isolation protocol to ensure methodological consistency. The isolated HSFs were cultured in high glucose Dulbecco’s modified Eagle’s medium (DMEM, Gibco) containing 10% fetal bovine serum (FBS, Gibco). NSFs were cultured under identical conditions using the same culture medium formulation. Cells were kept in a 37˚C incubator with 5% CO_2_ and passaged every 3–4 days. The characteristics of HSFs we isolated has been identified in our previous study^6^.

## Treatments

DSK (Cat. HY-N2187, 99.75% purity) was purchased from MedChemExpress (MCE, New Jersey, USA), which was dissolved in dimethylsulfoxide (DMSO) at a concentration of 10 mM as the primary stock and further diluted in medium to different concentration levels (100, 200, 500, 1000, and 2000 nM) for use. For phosphorylation analysis experiments (Fig. 7), HSFs were subjected to serum starvation in DMEM containing 0.5% fetal bovine serum for 12 h prior to drug treatments to minimize baseline phosphorylation levels. Following serum starvation, cells were treated with DSK or other reagents in serum-free medium.​ Chloroquine (CQ) is the inhibitor of autophagy. For the combined treatment of DSK and CQ (20 µM, Cat. HY-17589 A, MCE), HSFs were pre-treated with CQ for 6 h, followed by stimulation with DSK. PD98059 is an ERK1/2 inhibitor^20^. In pathway inhibition test, cells were pretreated with PD98059 (20 µM, Cat. HY-12028, MCE) for 1 h and then incubated with 1000 nM DSK.

## Cell infection

For FBXO2 overexpression and Atg5 knock-down, the FBXO2 overexpressed (oe FBXO2), empty (oeNC) and the specific Atg5 shRNA (sh- Atg5), scrambled control (sh-NC) lentiviruses were designed and synthesized by GenePharma Pharmaceutical Technology Co., Ltd (Shanghai, China). A multiplicity of infection (MOI) of 50 was utilized for HSFs lentivirus infection. At 3 days after infection, the transfection efficiency was validated by western blot, and the infected cells were treated with 1µM DSK for 24 h.

## Cell Counting Kit (CCK)−8 assay

The cell viability was detected by CCK-8 assay. HSFs or infected cells were treated with different concentrations of DSK (0, 100, 200, 500, 1000, and 2000 nM) and were added into 96-well plates (1000 per well). After incubation for 24 h, 10 µL of CCK-8 solution (Beyotime, Shanghai, China) was added into each well. The optical density (OD) value was evaluated at 450 nm.

**5-Ethynyl-20-deoxyuridine (Edu) assay**.

HSFs or infected cells treated with or without 1000 nM DSK were inoculated in 24-well plates for culture. When the cell density reached 60%−70%, the medium was removed and EdU (Beyotime) was added for 2 h. The cells were then stained and photographed under a microscope.

## Flow cytometric analysis of cell apoptosis

HSFs or infected cells after different treatments were detached by recombinant trypsin–EDTA solution, a single-cell suspension was prepared in PBS at a concentration of 1 × 10^6^ cells/mL, and doubly stained with 5 µl Annexin V-FITC and propidium iodide (PI, Beyotime) in the dark for 15 min at room temperature. Then apoptosis signals were detected by flow cytometer (BD Biosciences, CA, USA).

### Autophagy flux analysis

HSFs were infected with mRFP-GFP‐LC3 adenovirus (Hanbio, Shanghai, China) and cultured for 24 h. Then, the cells were treated with 1000 nM DSK or negative control for another 12 h. Subsequently, the cells were fixed with paraformaldehyde (PFA) and nuclei were stained with DAPI (Sigma). Finally, the images of cells were taken using fluorescence microscopy.

## Immunofluorescence staining

HSFs after different treatments were fixed with 4% paraformaldehyde for 10 min and then permeabilized with 0.05% Triton X-100 for 10 min. Non-specific binding sites were blocked with 5% goat serum for 1 h. Subsequently, the cells were incubated with polyclonal antibody to FBXO2 (#C02364F, SAB, USA) at room temperature for 1.5 h. After washing three times with PBS, the cells were incubated with FITC-conjugated goat anti-rabbit IgG (#7074, CST, USA) for 2 h at room temperature. Finally, positive cells were observed and quantified using a fluorescence microscope.

## Western blot analysis

Protein samples from HSFs and infected cells were extracted using Minute Total Protein Extraction Kit (Invent Biotechnologies, USA). The content of each sample was determined by BCA protein assay kit (Beyotime). Subsequently, the proteins were separated and transferred to PVDF membranes (Millipore, USA). Following by blocking with 5% skimmed milk, the membranes were incubated with primary antibodies specific to B-cell lymphoma protein 2 (Bcl-2, 1:1000, #3498, CST, USA), BCL2-associated X protein (Bax, 1:1000, #2772, CST), cleaved caspase-3 (1:1000, #9661, CST), cleaved poly ADP-ribose polymerase (PARP, 1:1000, #9541, CST), FBXO2 (1:10000, ab133717, Abcam, USA), microtubule-associated protein 1 light chain 3 (LC3, 1:1000, #12741, CST), P62 (1:1000, #39749, CST), Atg5 (1:1000, #12994, CST), collagen type I (1:1000, #ab34710, Abcam, USA), collagen type III (1:1000, #ab6310, Abcam), α-smooth muscle actin (α-SMA, 1:1000, #ab5694, Abcam), fibronectin (1:1000, #ab2413, Abcam), P38 (1:1000, #9212, CST), phosphorylated (p)-P38 (1:1000, #4511, CST), extracellular regulated kinase 1/2 (ERK1/2, 1:1000, #9102, CST), p-ERK1/2 (1:1000, #4370, CST), c-Jun N-terminal kinase (JNK, 1:1000, #9252, CST), p-JNK (1:1000, #4668, CST), and β-tubulin (1:1000, #2146, CST). Then, the membranes were incubated with HRP-linked goat anti-rabbit IgG secondary antibodies (1:3000, # 7074, CST). ECL (Millipore) solution was used to observe the protein band. Relative protein expression was quantified by BioImaging Systems.

### Co-immunoprecipitation assay (Co-IP)

The interaction between P62 and FBXO2 in HSFs was examined using a Co-IP assay. Following treatment, HSFs were lysed using the IP buffer. Lysates were precleared with protein A/G agarose beads and incubated overnight with primary antibodies against P62 (CST) and FBXO2 (Abcam). IgG was used as a negative control. The antibody-protein complexes were captured with fresh beads, and separated via SDS-PAGE and transferred onto PVDF membranes. Western blotting was performed using specific primary antibodies followed by HRP‐conjugated secondary antibodies.

### Statistical analysis

SPSS 23.0 was used to analyze the data. All experiments were performed in triplicate (*n* = 3 independent experiments) and measurement data were expressed as mean ± SD with individual data points shown in the figures. Prior to statistical analysis, data normality was assessed using the Shapiro-Wilk test and homogeneity of variances was evaluated using Levene’s test. The differences among groups were evaluated by one-way ANOVA followed by Tukey’s honestly significant difference (HSD) post hoc test for multiple comparisons. Student’s t-test was used for comparison between two groups when data met the assumption of normal distribution. *p* < 0.05 was considered statistically significant.

## Results

### DSK inhibited the proliferation of HSFs

**Fig. 1:**
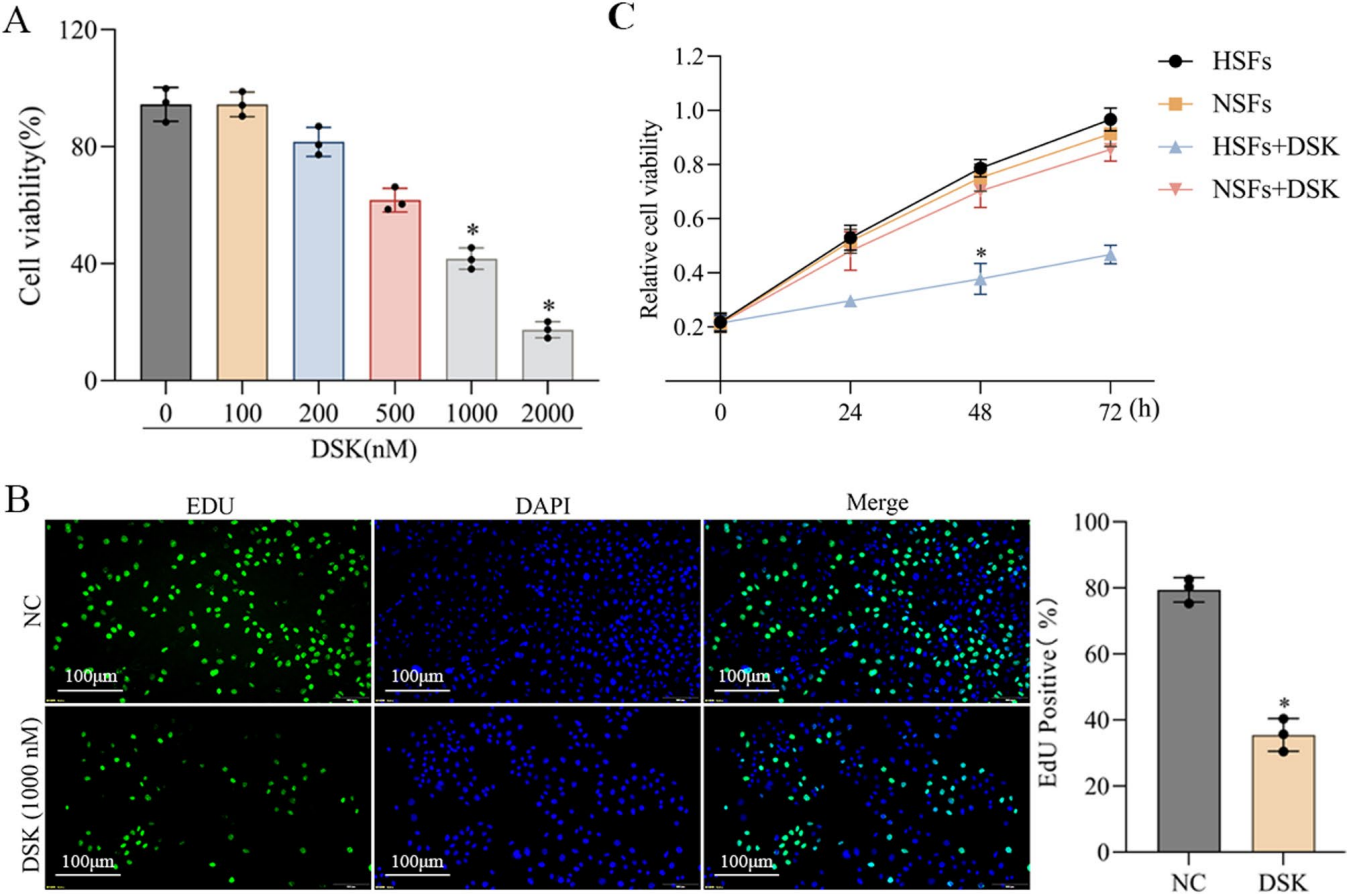
Deoxyshikonin (DSK) inhibited the proliferation of hypertrophic scar-derived fibroblasts (HSFs). HSFs were treated with different concentrations of DSK (0, 100, 200, 500, 1000, and 2000nM), and the cell viability was detected by CCK-8 assay (A). HSFs were treated with or without 1000nM DSK, and the proliferation of HSFs was detected by EdU assay (B). Comparison of the cell viability of HSFs and NSFs by DSK(C). Data are presented as mean ± SD from three independent experiments (*n* = 3) with individual data points shown. *P < 0.05 vs. NC group. Scale bar: 100 μm.

In order to investigate whether DSK had capability to inhibit the cell proliferation of HSFs. HSFs were treated with different concentrations of DSK (0, 100, 200, 500, 1000, and 2000 nM), and the cell viability was detected by CCK-8 assay. Our results indicated that DSK significantly inhibited the viability of HSFs in a dose-dependent manner (Fig. 1A). In addition, the EdU assay further demonstrated that the proliferation of HSFs was observably attenuated after DSK treatment (Fig. 1B). To evaluate the selectivity of DSK for pathological cells, we compared the effects of DSK on HSFs and NSFs. The results showed that at the same concentration, the inhibitory effect of DSK on HSFs was significantly stronger than that on NSFs (Fig. 1C).Taken together, the above findings suggested that DSK treatment could suppress the proliferation of HSFs.

### DSK induced the apoptosis of HSFs

**Fig. 2:**
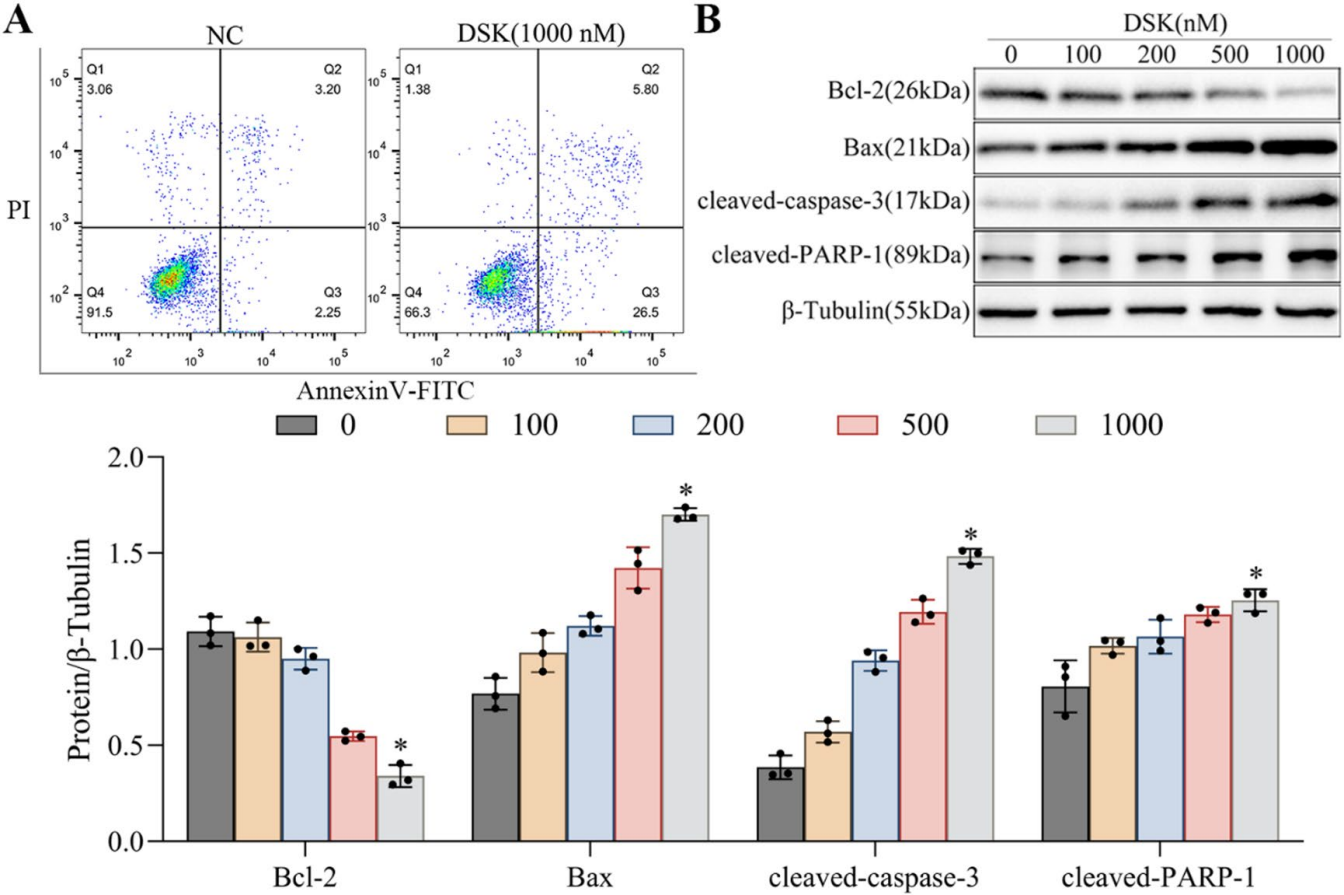
DSK induced the apoptosis of HSFs. HSFs were exposed to DSK for 24 h, and the evaluation of the apoptotic ratio was performed using flow cytometry(A). The effect of DSK on apoptosis-associated proteins was examined by western blot(B). Data are presented as mean ± SD from three independent experiments (*n* = 3) with individual data points shown. *P < 0.05 vs. NC group.

Previously, DSK has been shown to trigger apoptosis in tumor cells^21,22^. To determine whether the growth inhibition effect of DSK on HSFs resulted from apoptosis, HSFs were exposed to DSK for 24 h. Evaluation of the apoptotic ratio was performed using flow cytometry. The results showed that DSK induced an increase in the percentage of apoptotic cells, as comparing to the control (Fig. 2A). To further confirm these findings, the effect of DSK on apoptosis-associated proteins was examined by western blot. We found that DSK dose-dependently increased the expressions of Bax, cleaved caspase-3 and cleaved PARP, and decreased the expression of apoptosis-inhibitory protein Bcl-2 (Fig. 2B). Furthermore, Western blot analysis demonstrated that DSK treatment significantly downregulated the expression of key ECM components, including collagen type I, collagen type III, α-SMA, and fibronectin (S1), indicating the multi-faceted anti-fibrotic effects of DSK.The above findings indicated that DSK treatment could induce the apoptosis of HSFs.

### DSK inhibits the expression of FBXO2 in HSFs

**Fig. 3:**
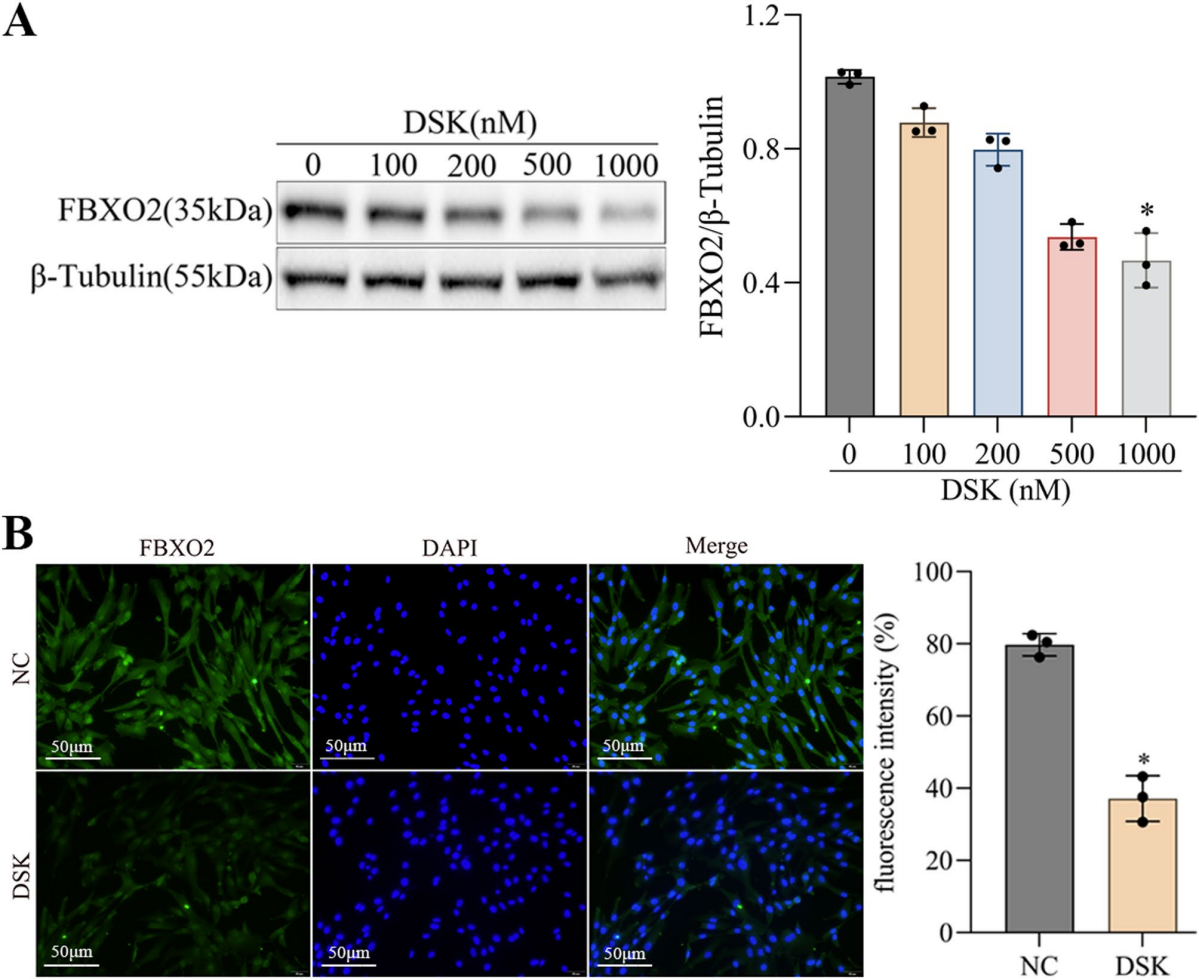
DSK inhibits the expression of FBXO2 in HSFs. HSFs were treated with different concentrations of DSK (0, 100, 200, 500 and 1000nM) and the expressions of FBXO2 were evaluated by western blot (A). The immunofluorescence staining confirmed that DSK decreased FBXO2 expression at a protein level, HSFs were treated with 1000 nM DSK for 24 h prior to immunofluorescence staining (B). Data are presented as mean ± SD from three independent experiments (*n* = 3) with individual data points shown. Scale bars: 50 μm *P < 0.05 vs. NC group.

Previous studies have showed that F-box proteins were upregulated in hypertrophic scars and keloid tissues, which promoted fibroblast growth^23^. FBXO2 belongs to the family of F-box proteins. Regulating FBXO2 may contribute to the inhibition of HSFs growth. To validate whether DSK could regulate FBXO2 expression, HSFs were treated with different concentrations of DSK (0, 100, 200, 500 and 1000 nM) and the expressions of FBXO2 were evaluated by western blot. The western blot showed that DSK dose‐dependently down-regulated the expression of FBXO2 (Fig. 3A). Furthermore, the immunofluorescence staining confirmed that DSK decreased FBXO2 expression at a protein level (Fig. 3B). DSK inhibited the expression of FBXO2 in HSFs, as evidenced by decreased protein levels detected via Western blot and immunofluorescence staining.

### FBXO2 overexpression reverses the role of DSK on proliferation and apoptosis in HSFs

**Fig. 4:**
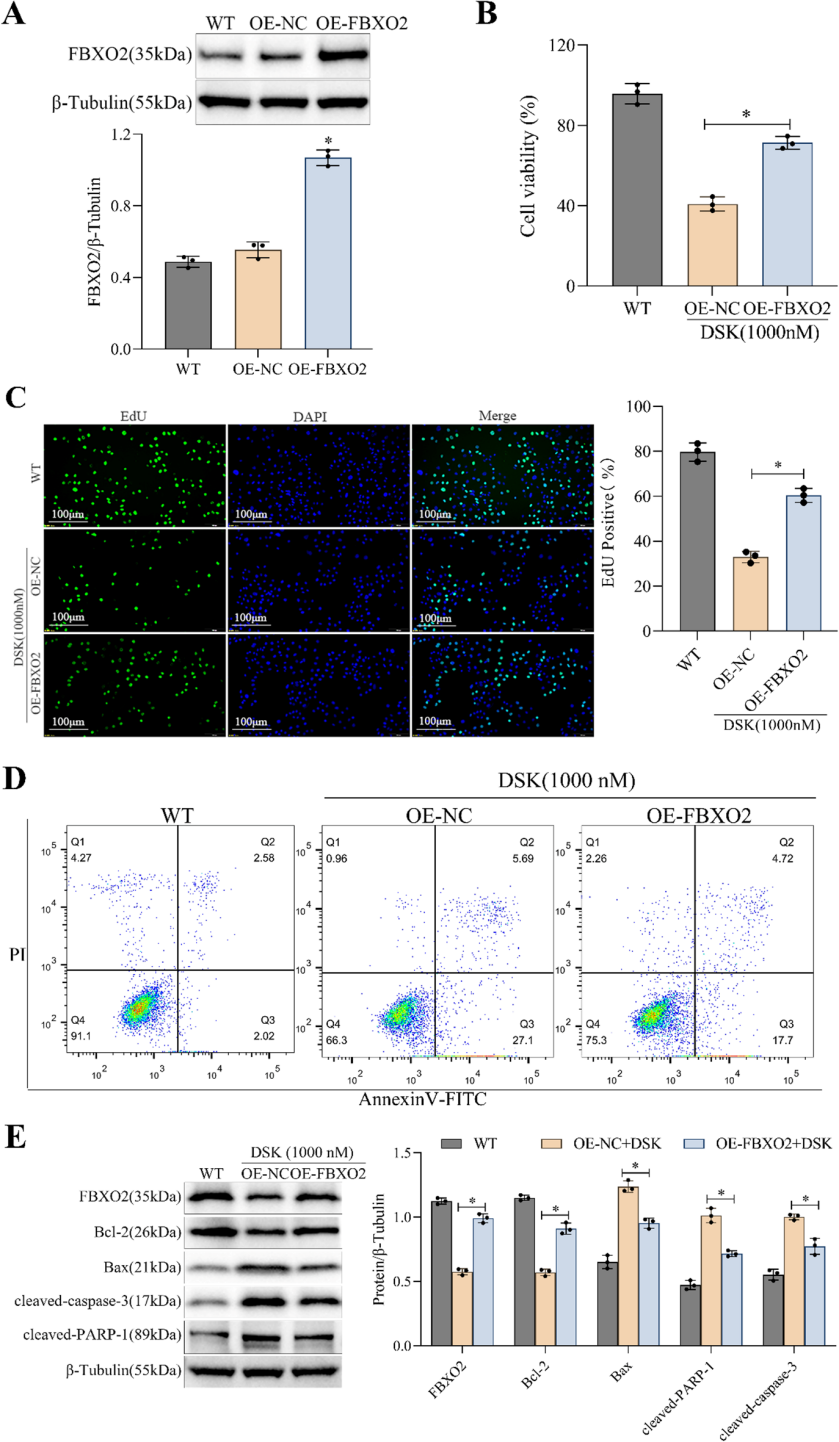
DSK limits the proliferation of HSFs by inhibiting FBXO2 expression. HSFs were infected with oeNC and oeFBXO2 lentivirus before treated with DSK. The transfection efficiency was validated by western blot (A). The cell viability was detected by CCK-8 assay (B). The proliferation of HSFs was detected by EdU assay (C). The cell apoptosis rate was evaluated by flow cytometry analysis(D). The expression of apoptosis-associated proteins was detected by western blot (E). Data are presented as mean ± SD from three independent experiments (*n* = 3) with individual data points shown. Scale bars: 100 μm *P < 0.05 vs. OE-NC + DSK group.

We next investigated whether DSK inhibited the growth of HSFs through regulating FBXO2 expression. To achieve this purpose, HSFs were infected with oeNC and oeFBXO2 lentivirus and the infective efficiency was validated by western blot. We found that oeFBXO2 significantly increased FBXO2 expression in HSFs (Fig. 4A). Then, the infected cells were treated with or without 1000nM DSK and the cell proliferation was detected by CCK-8 and Edu assays. The findings showed that oeFBXO2 markedly reduced the inhibitory effect of DSK treatment on cell proliferation​ compared with the oeNC + DSK group (Fig. 4B-C, *P* < 0.05). For cell apoptosis, flow cytometry analysis showed that oeFBXO2 partially reduced DSK-induced apoptosis​ in HSFs (Fig. 4D, *P* < 0.05). Furthermore, in DSK-treated cells, oeFBXO2 resulted in lower levels of Bax, cleaved caspase-3 and cleaved PARP, and higher levels of Bcl-2, comparing to oeNC (Fig. 4E). The above results suggested that DSK inhibited HSFs proliferation and induced HSFs apoptosis by regulating FBXO2 expression.

### DSK induced autophagy in HSFs

**Fig. 5:**
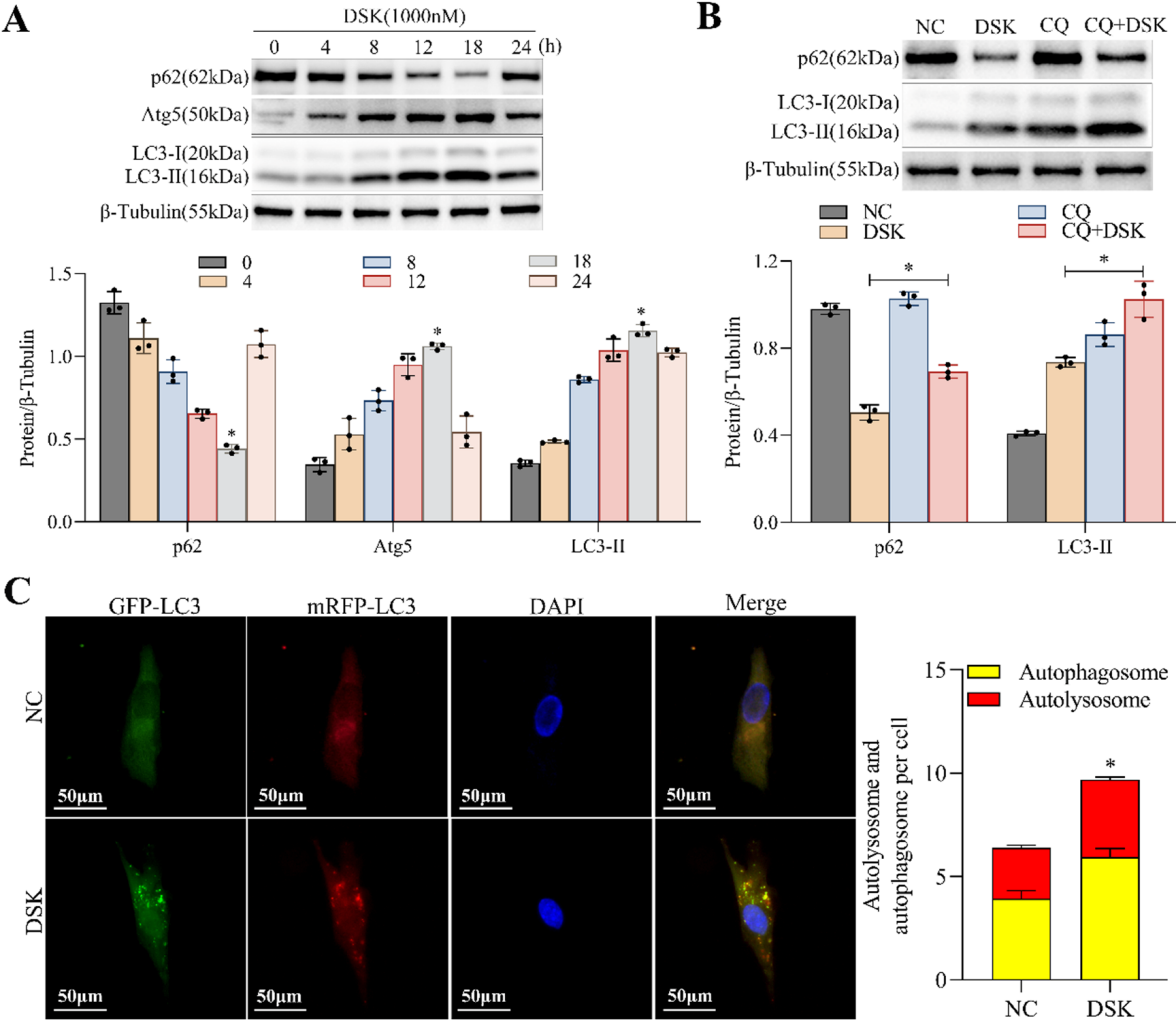
DSK induced autophagy in HSFs. HSFs were treated with 1000nM DSK for different time (0, 4, 8, 12, 18, and 24 h), and the expression of LC3 was detected by western blot, *P < 0.05 vs. NC group (A). HSFs were treated with DSK and/or CQ, and the expressions of LC3 and P62 were evaluated by western blot analysis, *P < 0.05 vs. DSK group (B). The formation of autophagosomes was observed by mRFP-GFP-LC3 adenovirus double label assay, *P < 0.05 vs. NC group (C). Data are presented as mean ± SD from three independent experiments (*n* = 3) with individual data points shown. Scale bars: 50 μm.

In a previous study, DSK has been reported to regulate autophagy in rotavirus^10^. In this study, we hypothesized that DSK might be involved in autophagy activation in HSFs. To validate our hypothesis, HSFs were treated with 1000nM DSK for different time periods (0, 4, 8, 12, 18, and 24 h), and the expression levels of key autophagy markers including LC3, Atg5 and p62 were detected by western blot. We found that DSK treatment induced a time-dependent increase in LC3-II and Atg5 protein levels, accompanied by a decrease in p62 expression (Fig. 5A). The coordinated changes in these autophagy markers provide direct evidence for DSK-sustained complete autophagic flux, with the most pronounced effects observed at the 18-hour time point. After that, HSFs were treated with DSK and/or CQ, and the expressions of LC3 and P62 were evaluated by western blot analysis. CQ can block the autophagic flux at late stage by inhibiting the fusion with lysosomes. Compared with DSK treatment group, western blot showed that DSK plus CQ group further increased the ratio of LC3II/LC3I and protein expression of P62 in HSFs (Fig. 5B). In addition, the formation of autophagosomes was observed by mRFP-GFP-LC3 adenovirus double label assay. We found that DSK increased the number of cytoplasmic autophagosomes comparing to the control (Fig. 5C). The above findings suggested that DSK possessed the ability to induce autophagy in HSFs.

### DSK suppresses FBXO2 expression in HSFs through autophagy

**Fig. 6:**
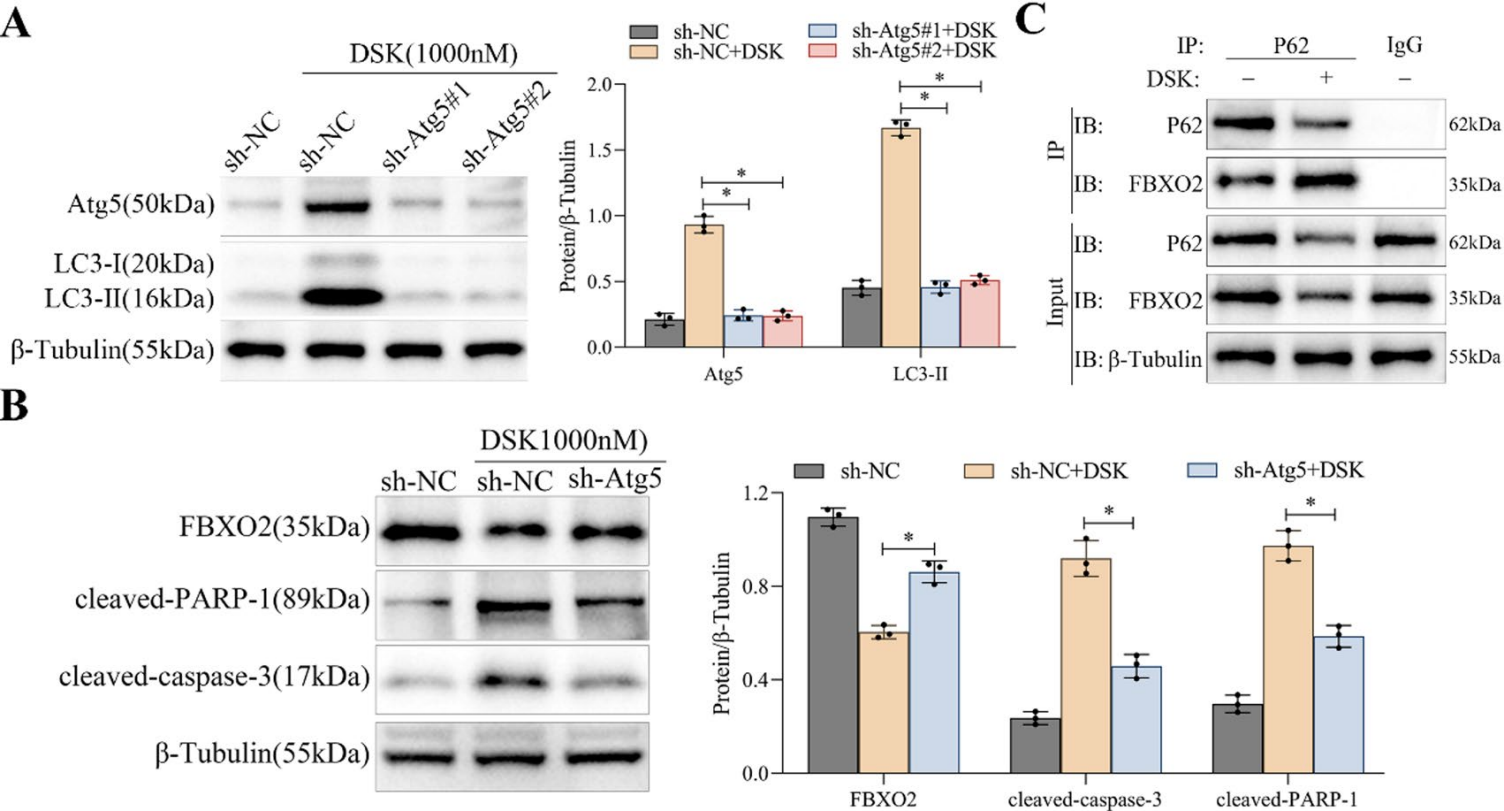
DSK induced autophagy mediates FBXO2 degradation in HSFs. HSFs infected with sh-Atg5 or sh-NC were treated with 1000 nM DSK for 18 h, and the expression of autophagy-related proteins was detected by western blot. The time point was selected based on our previous optimization showing maximal autophagic flux at 18 h (A). The expression of FBXO2 and apoptosis-associated proteins in HSFs infected with sh-Atg5 or sh-NC after treatment with 1000 nM DSK for 18 h​ were evaluated by western blot. The 18-hour time point was chosen to assess apoptosis-related protein changes. (B). The interaction between P62 and FBXO2 in HSFs treated with 1000 nM DSK for 18 h​ was validated by co-immunoprecipitation assay. This time point corresponds to the peak of autophagic activity observed in our time-course experiments (C). Data are presented as mean ± SD from three independent experiments (*n* = 3) with individual data points shown. *P < 0.05.

Previously, a prominent role of autophagy on targeted protein degradation has been established^24^. To establish the functional role of the regulatory relationship between DSK and FBXO2 expression in the autophagic program of HSFs, sh-Atg5 was used to inhibit the autophagy in HSFs. Compared with control shRNA, Atg5 knockdown reduced Atg5 and LC3II expression in DSK-treated HSFs (Fig. 6A). Then, the expressions of FBXO2 and apoptosis-associated proteins after sh-Atg5 infection were evaluated by western blot. We found that sh-Atg5 resulted in increased expressions of FBXO2 and decreased expressions of cleaved caspase-3 and cleaved PARP in DSK-treated HSFs, comparing to control shRNA (Fig. 6B). Finally, we verified whether there was an interaction between autophagic adaptor protein P62 and FBXO2 by a co-immunoprecipitation assay. Our results demonstrated a direct interaction between P62 and FBXO2 (Fig. 6C). The above findings indicated that DSK mediated the degradation of FBXO2 through autophagy pathway.

### DSK induced autophagy and FBXO2 degradation regulated via activating ERK1/2 pathway in HSFs

**Fig. 7:**
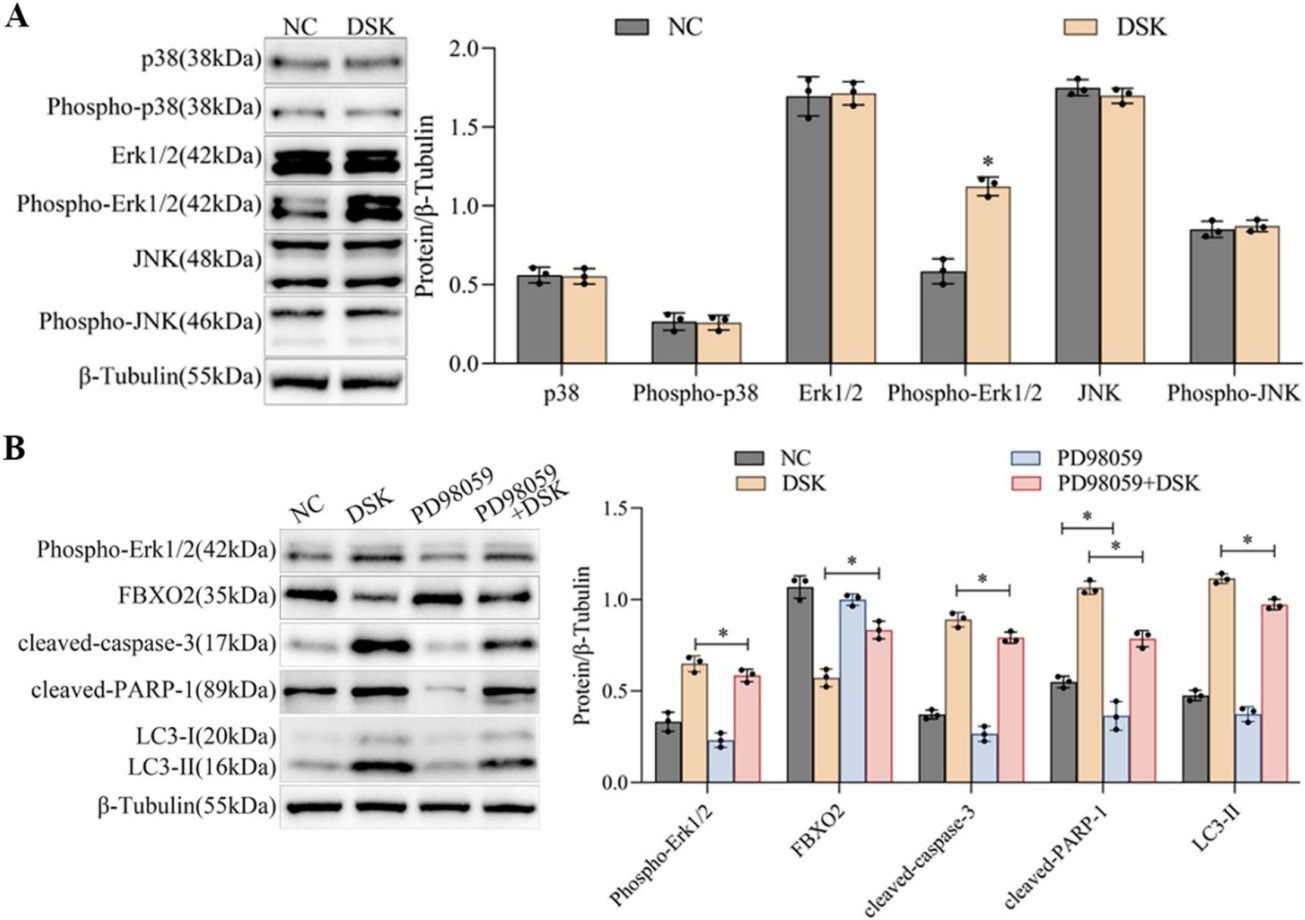
DSK induced autophagy and FBXO2 degradation in HSFs by activating ERK1/2 signaling. HSFs were treated with or without DSK for 30 min, and the expressions of proteins in MAPK pathway were detected by western blot (**A**). HSFs were treated with DSK and/or PD98059 for 24 h, followed by western blot analysis to detect the expressions of autophagic markers, apoptosis-associated proteins and FBXO2 (**B**). Data are presented as mean ± SD from three independent experiments (*n* = 3) with individual data points shown. *P < 0.05.

Mitogen-activated protein kinase (MAPK) signaling pathway has been demonstrated to participate in the proliferation of fibroblasts in HS^25^. It is well known that the molecules involved in MAPK family mainly include JNK, ERK1/2, and p38^26^. Our western blot analysis showed that DSK did not affect the expressions of p-P38, P38, p-JNK and JNK in HSFs (Fig. 7A). However, p-ERK1/2 levels were significantly increased after DSK treatment (Fig. 7A), indicating DSK could regulate the phosphorylation of ERK1/2. PD98059 is an ERK1/2 inhibitor. HSFs were treated with DSK, PD98059, DSK plus PD98059 or negative control for 24 h, followed by western blot to detect the expression of autophagic markers, apoptosis-associated proteins and FBXO2. We found that PD98059 showed an antagonistic action against DSK to decrease the expressions of LC3II, cleaved caspase-3 and cleaved PARP, and increase the expressions of FBXO2 (Fig. 7B). Taken together, our findings demonstrated that DSK downregulated the expression of FBXO2 in HSFs by autophagy via ERK1/2 signaling.

## Discussion

HS is a fibro-proliferative disorder of cutaneous wound healing, which seriously impacts the quality of patient’s life. The formation of HS is mainly caused by abnormal proliferation and migration of HSFs^13^. Inhibiting HSF proliferation is a key process to control HS. Previously, the inhibitory effects of shikonin on HSFs have been identified^13^. Thus, it is possible for DSK to inhibit HS derived fibroblasts growth by which promotes cutaneous wound healing. In the present study, DSK was demonstrated to induce apoptosis and inhibit the proliferation of HSFs. Mechanically, we found the proliferation and apoptosis of HSFs was determined by DSK induced autophagy by which promoted degradation of FBXO2 via the activation of ERK1/2 signaling pathway. Our study demonstrated for the first time that anti-fibrotic functions of DSK in HSFs, providing a novel strategy to improve the therapeutic effects of HS.

The balance between cell survival and cell death is necessary for maintaining tissue homeostasis^13^. Apoptosis is an important form of programmed cell death. Previous studies have shown that drugs triggering the apoptosis of HSFs could inhibit HS formation^6,27^. As DSK has been reported to trigger cancer cell apoptosis^21,22^, we hypothesized that DSK could also induce apoptosis of HSFs. As expected, flow cytometry analysis found that DSK induced an increase in the percentage of apoptotic cells. Apoptosis is regulated by several proteins including Bcl-2 family proteins (e.g. Bcl-2, Bax), caspase family proteins (e.g. caspase-3), and PARP^28^. During the process of apoptosis, the increased levels of Bax, cleaved caspase-3 and cleaved PARP, and down-regulation of Bcl-2 can be observed. In the present study, we found that DSK increased the expressions of pro-apoptotic proteins and decreased the expression of anti-apoptotic protein. The above findings indicated that DSK could suppress HSFs cells proliferation by inducing the apoptosis.

F-box proteins regulate substrates in diverse biologic processed that control essential aspects of cellular life, including cell growth, cell survival and death^29^. FBXO2, a well-known E3 ligase, is a cytoplasmic protein belongs to the family of F-box proteins. It mediates the ubiquitination of endoplasmic reticulum (ER) glycoproteins in the ER-associated degradation system, playing an important role in metabolic diseases, Parkinson’s disease and tumor development^30^. In hypertrophic scars and keloid tissues, F-box protein family member FBXL6 has been reported to promote fibroblast growth^23^. Thus, regulating F‐box protein family members may contribute to HS and keloids treatments. To validate whether FBXO2 participated in the regulation role of DSK on HSFs, we firstly investigated the effect of DSK on FBXO2 expression. As expected, we found that DSK significantly suppressed the expression of FBXO2 in a dose-dependent manner. Subsequently, we utilizing oeFBXO2 lentivirus to overexpress FBXO2 and observed the effects of FBXO2 on DSK-treated HSFs cells. We found that oeFBXO2 abolished the effects of DSK treatment on cell proliferation and apoptosis. These results suggested that FBXO2 participated in the regulation role of DSK on HSFs.

Autophagy, an intracellular self-degradative mechanism, has been reported to be tightly associated with the survival, differentiation, and maintenance of fibroblasts during the wound healing process^30^. Some papers even pointed out that the dysregulation of autophagy was a pathological basis of HS formation^30,31^. Thus, targeting autophagy might be a promising approach to treat HS. More importantly, DSK has been shown to regulate autophagy in rotavirus^10^. Thus, it is rational to hypothesize that DSK has the potential to modulate autophagy in HSFs. As expected, our study indicated that DSK contributed to observable elevation of LC3-II in HSFs, accompanied with accumulation of autophagosomes. Using the CQ to interupt autophagy flux, the protein level of LC3-II and P62 were further increased in HSFs under DSK stimulation. Taken together, our findings confirmed that DSK had capability to trigger autophagy in HSFs.

Atg5 is a core autophagy protein, regulating the formation of the autophagosome^32^. Knocking down or knocking out Atg5 can result in downregulation or total inhibition of autophagy^33^. In the present study, sh-Atg5 was used to knockdown Atg5 and inhibit autophagy. We found that the effects of DSK on the expressions of FBXO2 and apoptosis-associated proteins were successfully abolished by Atg5 knockdown. P62 has been identified as one of the specific substrates that are degraded during the process of autophagy^34^. Using co-immunoprecipitation assay, we demonstrated a direct interaction between P62 and FBXO2. It has been reported that intracellular proteins can be degraded via the autophagy-lysosome pathway^35^. Our findings demonstrated that DSK mediated FBXO2 degradation through autophagy pathway.

Furthermore, our supplementary experiments revealed that DSK not only induces apoptosis in HSFs but also significantly inhibits the deposition of key extracellular matrix (ECM) components. As shown in Supplementary Fig. 1, DSK treatment markedly downregulated the expression levels of collagen type I, collagen type III, α-smooth muscle actin (α-SMA), and fibronectin. This finding provides direct evidence for the anti-fibrotic efficacy of DSK beyond its pro-apoptotic effects.From a mechanistic perspective, the inhibitory effect of DSK on ECM deposition appears to be closely associated with its regulation of the autophagy pathway. Previous studies have demonstrated that autophagy activation can promote the degradation of abnormally deposited ECM components, and our experimental results support this viewpoint. DSK may facilitate the clearance of fibrosis-related proteins through activating the autophagic pathway, thereby alleviating excessive ECM accumulation.Notably, the ‘dual-action’ mechanism demonstrated by DSK—simultaneously reducing the number of fibrotic effector cells and inhibiting excessive ECM synthesis—confers unique advantages for HS treatment. This synergistic mechanism holds promise for providing new insights into clinical HS therapy.

MAPK signaling pathway has been demonstrated to participate in the proliferation of fibroblasts in HS^25^. It is well known that the key proteins of the MAPK family involved in inflammation mainly include JNK, ERK1/2, and p38^26^. Previous studies revealed that DSK induced wound healing and triggered apoptosis of cancer cells through the activation of ERK1/2 pathway^21,36^. In the present study, we also found that DSK upregulated the phosphorylation of MAP kinase protein ERK1/2, but not p38 and JNK. Previously, ERK1/2 activation has been found in skin fibroblasts, which plays an important role in fibroblasts growth^37,38^. Furthermore, ERK1/2 signaling is also closely related to the process of autophagy^39^. For instance, Kim et al.^40^ found that abnormal activation of ERK1/2 increased the expression of important autophagy markers such as LC3II in LNCaP cells, HEK293 cells, BJ cells, and IMR90E1A cells. Chen et al.^41^ also revealed that the ERK1/2 signaling pathway played an important role in inhibiting the expression of Cathepsin S to promote autophagy in HONE 1 cells. In this study, using PD98059 to inhibit ERK1/2 signaling, we found that the effects of DSK on FBXO2 expression, apoptosis and autophagy were alleviated as expected. Thus, our study confirmed that DSK downregulated the expression of FBXO2 in HSFs by autophagy via ERK1/2 signaling pathway.

Although this study offers novel insights into the molecular mechanisms underlying DSK’s anti‑fibrotic effects, it is important to emphasize that our findings are derived exclusively from in vitro experiments. The complex in vivo scar microenvironment involves dynamic interactions among fibroblasts, immune cells, vascular elements, and extracellular matrix components, which cannot be fully recapitulated in cell culture. Consequently, future investigations using well‑designed animal models will be critical to validate these results and to assess the translational potential of DSK for clinical use. In addition, while we have demonstrated that FBXO2 modulates DSK-induced changes in proliferation, apoptosis, and autophagy, the downstream molecular pathways and broader signaling networks involved remain incompletely defined; integrated approaches such as transcriptomics and proteomics will be required to map these interactions comprehensively. Finally, our experiments were primarily based on short‑term treatments (24–48 h), and comprehensive long‑term functional and safety evaluations are lacking. Systematic optimization of concentration gradients is also needed to better balance therapeutic efficacy with potential toxicity. Together, these limitations highlight clear directions for future research, including in vivo validation, deeper mechanistic dissection, and refined dosing strategies.

In conclusion, our findings firstly demonstrate that autophagy induced by DSK confound the proliferation and promoted apoptosis of HSFs cells through increasing the degradation of FBXO2 via activating ERK1/2 signaling. Therefore, DSK provides a novel potential treatment for HS, and modulating autophagy may provide a putative method to elevate therapeutic outcome of DSK in HS.

## Supplementary Information

Below is the link to the electronic supplementary material.

Supplementary Material 1

Supplementary Material 2

Supplementary Material 3

Supplementary Material 4

## Data Availability

The data that support the findings of this study are available from the corresponding author upon reasonable request.

## References

[1] Li, Y. et al. Exosomes derived from human adipose mesenchymal stem cells attenuate hypertrophic scar fibrosis by miR-192-5p/IL-17RA/Smad axis. *Stem Cell Res. Ther.* **12**, 221. 10.1186/s13287-021-02290-0 (2021). 33789737 10.1186/s13287-021-02290-0 PMC8010995

[2] Chen, D. et al. Traditional Chinese medicine for hypertrophic scars-A review of the therapeutic methods and potential effects. *Front. Pharmacol.* **13**10.3389/fphar.2022.1025602 (2022). 10.3389/fphar.2022.1025602 PMC9589297 36299876

[3] Wang, Z. C. et al. The Roles of Inflammation in Keloid and Hypertrophic Scars. *Front. Immunol.* **11**, 603187. 10.3389/fimmu.2020.603187 (2020). 33343575 10.3389/fimmu.2020.603187 PMC7746641

[4] Bi, M., Sun, P., Li, D., Dong, Z. & Chen, Z. Intralesional Injection of Botulinum Toxin Type A Compared with Intralesional Injection of Corticosteroid for the Treatment of Hypertrophic Scar and Keloid: A Systematic Review and Meta-Analysis. *Med. Sci. monitor: Int. Med. J. experimental Clin. Res.* **25**, 2950–2958. 10.12659/msm.916305 (2019). 10.12659/MSM.916305 PMC6489528 31006769

[5] Yin, J. et al. Mechanotransduction in skin wound healing and scar formation: Potential therapeutic targets for controlling hypertrophic scarring. *Front. Immunol.* **13**10.3389/fimmu.2022.1028410 (2022). 10.3389/fimmu.2022.1028410 PMC9618819 36325354

[6] Deng, X. et al. Oxymatrine promotes hypertrophic scar repair through reduced human scar fibroblast viability, collagen and induced apoptosis via autophagy inhibition. *Int. Wound J.* **19**, 1221–1231. 10.1111/iwj.13717 (2022). 34749441 10.1111/iwj.13717 PMC9284648

[7] Ning, X., Wiraja, C., Chew, W. T. S., Fan, C. & Xu, C. Transdermal delivery of Chinese herbal medicine extract using dissolvable microneedles for hypertrophic scar treatment. *Acta Pharm. Sinica B*. **11**, 2937–2944. 10.1016/j.apsb.2021.03.016 (2021). 10.1016/j.apsb.2021.03.016 PMC8463281 34589406

[8] Boulos, J. C., Rahama, M., Hegazy, M. F. & Efferth, T. Shikonin derivatives for cancer prevention and therapy. *Cancer Lett.* **459**, 248–267. 10.1016/j.canlet.2019.04.033 (2019). 31132429 10.1016/j.canlet.2019.04.033

[9] Kim, J. H. et al. Deoxyshikonin reversibly inhibits cytochrome P450 2B6. *Biopharm. Drug Dispos.* **41**, 221–225. 10.1002/bdd.2230 (2020). 32364297 10.1002/bdd.2230

[10] Huang, H. et al. Deoxyshikonin inhibited rotavirus replication by regulating autophagy and oxidative stress through SIRT1/FoxO1/Rab7 axis. *Microb. Pathog.* **178**, 106065. 10.1016/j.micpath.2023.106065 (2023). 36907361 10.1016/j.micpath.2023.106065

[11] Chuang, C. Y. et al. Deoxyshikonin Mediates Heme Oxygenase-1 Induction and Apoptotic Response via p38 Signaling in Tongue Cancer Cell Lines. *Int. J. Mol. Sci.* **23**10.3390/ijms23137115 (2022). 10.3390/ijms23137115 PMC9266306 35806120

[12] Debnath, J., Gammoh, N. & Ryan, K. M. Autophagy and autophagy-related pathways in cancer. *Nat. Rev. Mol. Cell Biol.* **24**, 560–575. 10.1038/s41580-023-00585-z (2023). 36864290 10.1038/s41580-023-00585-z PMC9980873

[13] Liu, Z., Chen, N. Y., Zhang, Z., Zhou, S. & Hu, S. Y. F-box only protein 2 exacerbates non-alcoholic fatty liver disease by targeting the hydroxyl CoA dehydrogenase alpha subunit. *World J. Gastroenterol.* **29**, 4433–4450. 10.3748/wjg.v29.i28.4433 (2023). 37576703 10.3748/wjg.v29.i28.4433 PMC10415968

[14] Shi, W., Wu, Y. & Bian, D. p75NTR silencing inhibits proliferation, migration, and extracellular matrix deposition of hypertrophic scar fibroblasts by activating autophagy through inhibiting the PI3K/Akt/mTOR pathway. *Can. J. Physiol. Pharmacol.* **99**, 349–359. 10.1139/cjpp-2020-0219 (2021). 32726570 10.1139/cjpp-2020-0219

[15] Pang, K. et al. Resveratrol inhibits hypertrophic scars formation by activating autophagy via the miR-4654/Rheb axis. *Mol. Med. Rep.* **22**, 3440–3452. 10.3892/mmr.2020.11407 (2020). 32945452 10.3892/mmr.2020.11407 PMC7453609

[16] Ji, J. et al. FBXO2 targets glycosylated SUN2 for ubiquitination and degradation to promote ovarian cancer development. *Cell Death Dis.* **13**10.1038/s41419-022-04892-9 (2022). 10.1038/s41419-022-04892-9 PMC9079088 35525855

[17] Fedorova, O. et al. Regulation of autophagy flux by E3 ubiquitin ligase Pirh2 in lung cancer. *Biochem. Biophys. Res. Commun.* **563**, 119–125. 10.1016/j.bbrc.2021.05.024 (2021). 34090148 10.1016/j.bbrc.2021.05.024

[18] Wang, P. et al. The F-box E3 ubiquitin ligase BAF1 mediates the degradation of the brassinosteroid-activated transcription factor BES1 through selective autophagy in Arabidopsis. *Plant. cell.* **33**, 3532–3554. 10.1093/plcell/koab210 (2021). 34436598 10.1093/plcell/koab210 PMC8566207

[19] Zhang, Q. et al. Shikonin promotes hypertrophic scar repair by autophagy of hypertrophic scar-derived fibroblasts. *Acta cirurgica brasileira*. **38**, e384623. 10.1590/acb384623 (2023). 37878984 10.1590/acb384623 PMC10592587

[20] Taguchi, K., Kaneko, N., Okudaira, K., Matsumoto, T. & Kobayashi, T. Endothelial dysfunction caused by circulating microparticles from diabetic mice is reduced by PD98059 through ERK and ICAM-1. *Eur. J. Pharmacol.* **913**, 174630. 10.1016/j.ejphar.2021.174630 (2021). 34774495 10.1016/j.ejphar.2021.174630

[21] Lee, C. Y. et al. Deoxyshikonin triggers apoptosis in cervical cancer cells through p38 MAPK-mediated caspase activation. *Environ. Toxicol.* **39**, 4308–4317. 10.1002/tox.24323 (2024). 38717057 10.1002/tox.24323

[22] Hsieh, M. C. et al. Apoptotic effect and cell arrest of deoxyshikonin in human osteosarcoma cells through the p38 pathway. *J. Cell. Mol. Med.* **27**, 1592–1602. 10.1111/jcmm.17764 (2023). 37155410 10.1111/jcmm.17764 PMC10243165

[23] Feng, G., Sun, H. & Piao, M. FBXL6 is dysregulated in keloids and promotes keloid fibroblast growth by inducing c-Myc expression. *Int. Wound J.* **20**, 131–139. 10.1111/iwj.13847 (2023). 35606330 10.1111/iwj.13847 PMC9797926

[24] Cozzi, M. & Ferrari, V. Autophagy Dysfunction in ALS: from Transport to Protein Degradation. *J. Mol. neuroscience: MN*. **72**, 1456–1481. 10.1007/s12031-022-02029-3 (2022). 10.1007/s12031-022-02029-3 PMC9293831 35708843

[25] Ma, F. et al. A novel lncRNA FPASL regulates fibroblast proliferation via the PI3K/AKT and MAPK signaling pathways in hypertrophic scar. *Acta Biochim. Biophys. Sin.* **55**, 274–284. 10.3724/abbs.2022122 (2022). 36082934 10.3724/abbs.2022122 PMC10157618

[26] Bai, R. J. et al. OPN inhibits autophagy through CD44, integrin and the MAPK pathway in osteoarthritic chondrocytes. *Front. Endocrinol.* **13**, 919366. 10.3389/fendo.2022.919366 (2022). 10.3389/fendo.2022.919366 PMC9411521 36034459

[27] Dong, Y. et al. Lycorine Inhibits Hypertrophic Scar Formation by Inducing ROS-Mediated Apoptosis. *Front. Bioeng. Biotechnol.* **10**, 892015. 10.3389/fbioe.2022.892015 (2022). 35685086 10.3389/fbioe.2022.892015 PMC9171077

[28] Zhou, L. et al. Lead acetate induces apoptosis in Leydig cells by activating PPARγ/caspase-3/PARP pathway. *Int. J. Environ. Health Res.* **31**, 34–44. 10.1080/09603123.2019.1625034 (2021). 31145012 10.1080/09603123.2019.1625034

[29] Che, X. et al. FBXO2 Promotes Proliferation of Endometrial Cancer by Ubiquitin-Mediated Degradation of FBN1 in the Regulation of the Cell Cycle and the Autophagy Pathway. *Front. cell. Dev. biology*. **8**, 843. 10.3389/fcell.2020.00843 (2020). 10.3389/fcell.2020.00843 PMC7487413 32984335

[30] Chen, N., Cao, R., Zhang, Z., Zhou, S. & Hu, S. Sleeve Gastrectomy Improves Hepatic Glucose Metabolism by Downregulating FBXO2 and Activating the PI3K-AKT Pathway. *Int. J. Mol. Sci.* **24**10.3390/ijms24065544 (2023). 10.3390/ijms24065544 PMC10052132 36982617

[31] Zeng, T. et al. Endothelial cell-derived small extracellular vesicles suppress cutaneous wound healing through regulating fibroblasts autophagy. *Clin. Sci. (London England: 1979)*. **133**10.1042/cs20190008 (2019). 10.1042/CS20190008 30988132

[32] Changotra, H. et al. ATG5: A central autophagy regulator implicated in various human diseases. *Cell Biochem. Funct.* **40**, 650–667. 10.1002/cbf.3740 (2022). 36062813 10.1002/cbf.3740

[33] Ye, X., Zhou, X. J. & Zhang, H. Exploring the Role of Autophagy-Related Gene 5 (ATG5) Yields Important Insights Into Autophagy in Autoimmune/Autoinflammatory Diseases. *Front. Immunol.* **9**, 2334. 10.3389/fimmu.2018.02334 (2018). 30386331 10.3389/fimmu.2018.02334 PMC6199349

[34] Komatsu, M. & Ichimura, Y. Physiological significance of selective degradation of p62 by autophagy. *FEBS Lett.* **584**, 1374–1378. 10.1016/j.febslet.2010.02.017 (2010). 20153326 10.1016/j.febslet.2010.02.017

[35] Paudel, R. R., Lu, D., Roy Chowdhury, S., Monroy, E. Y. & Wang, J. Targeted Protein Degradation via Lysosomes. *Biochemistry* **62**, 564–579. 10.1021/acs.biochem.2c00310 (2023). 36130224 10.1021/acs.biochem.2c00310 PMC10245383

[36] Park, J. Y. et al. Wound healing effects of deoxyshikonin isolated from Jawoongo: In vitro and in vivo studies. *J. Ethnopharmacol.* **199**, 128–137. 10.1016/j.jep.2016.10.031 (2017). 27725239 10.1016/j.jep.2016.10.031

[37] Kitanaka, N. et al. Interleukin-1β promotes interleulin-6 expression via ERK1/2 signaling pathway in canine dermal fibroblasts. *PloS one*. **14**, e0220262. 10.1371/journal.pone.0220262 (2019). 31344106 10.1371/journal.pone.0220262 PMC6658082

[38] Makino, T. et al. Basic fibroblast growth factor stimulates the proliferation of human dermal fibroblasts via the ERK1/2 and JNK pathways. *Br. J. Dermatol.* **162**, 717–723. 10.1111/j.1365-2133.2009.09581.x (2010). 19995368 10.1111/j.1365-2133.2009.09581.x

[39] Wang, L. et al. Netrin-1 regulates ERK1/2 signaling pathway and autophagy activation in wear particle-induced osteoclastogenesis. *Cell. Biol. Int.* **45**, 612–622. 10.1002/cbin.11544 (2021). 33386763 10.1002/cbin.11544 PMC8048890

[40] Kim, J. H. et al. Raf/MEK/ERK can regulate cellular levels of LC3B and SQSTM1/p62 at expression levels. *Exp. Cell Res.* **327**, 340–352. 10.1016/j.yexcr.2014.08.001 (2014). 25128814 10.1016/j.yexcr.2014.08.001 PMC4164593

[41] Chen, K. L. et al. Targeting cathepsin S induces tumor cell autophagy via the EGFR-ERK signaling pathway. *Cancer Lett.* **317**, 89–98. 10.1016/j.canlet.2011.11.015 (2012). 22101325 10.1016/j.canlet.2011.11.015

