# Supplementary material for: Deoxyshikonin inhibits growth and induces apoptosis of hypertrophic scar-derived fibroblasts by downregulating FBXO expression through autophagy
Source: Sci Rep. 2026 Apr 28;16:19700. doi: 10.1038/s41598-026-49808-1 (PMC13315721; doi:10.1038/s41598-026-49808-1)
Supplement: Supplementary file 1 — Supplementary Material 1 [file 41598_2026_49808_MOESM1_ESM.docx]

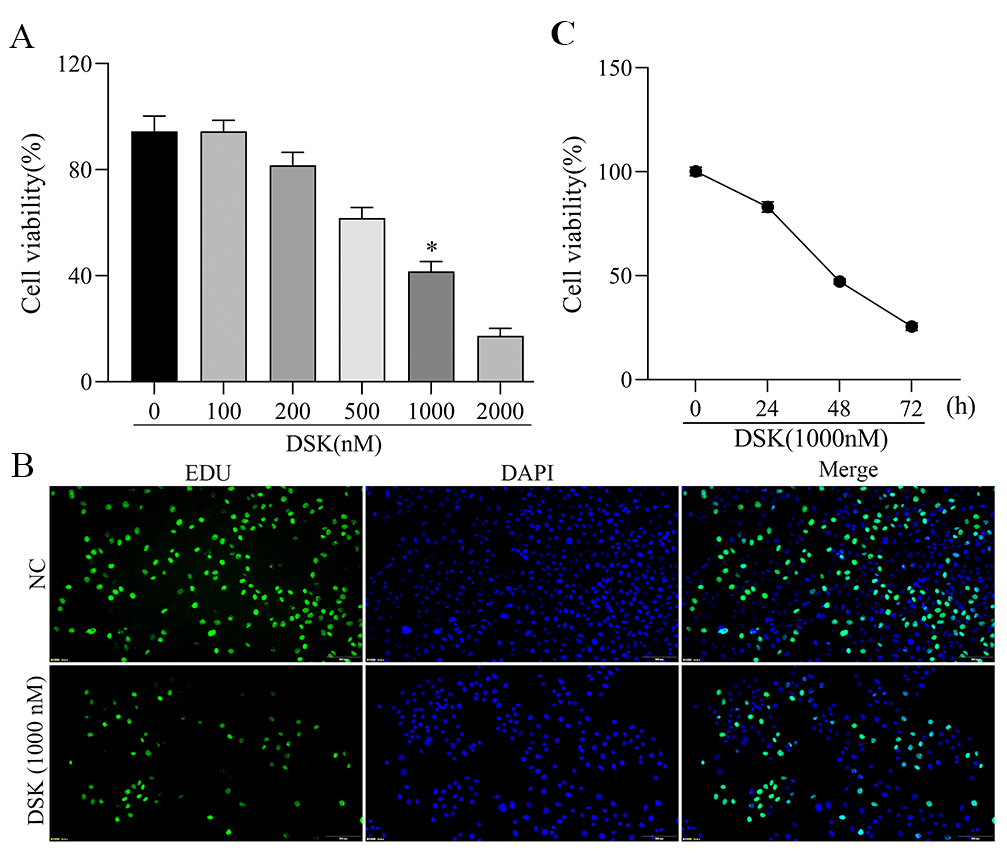


**Fig. 1.** Deoxyshikonin (DSK) inhibited the proliferation of hypertrophic scar-derived fibroblasts (HSFs). HSFs were treated with different concentrations of DSK (0, 100, 200, 500, 1000, and 2000nM), and the cell viability was detected by CCK-8 assay(A). HSFs were treated with or without 1000nM DSK, and the proliferation of HSFs was detected by EdU assay(B). Representative data from three separate experiments are shown. Scale bars: 100 µm **P* < 0.05.

**
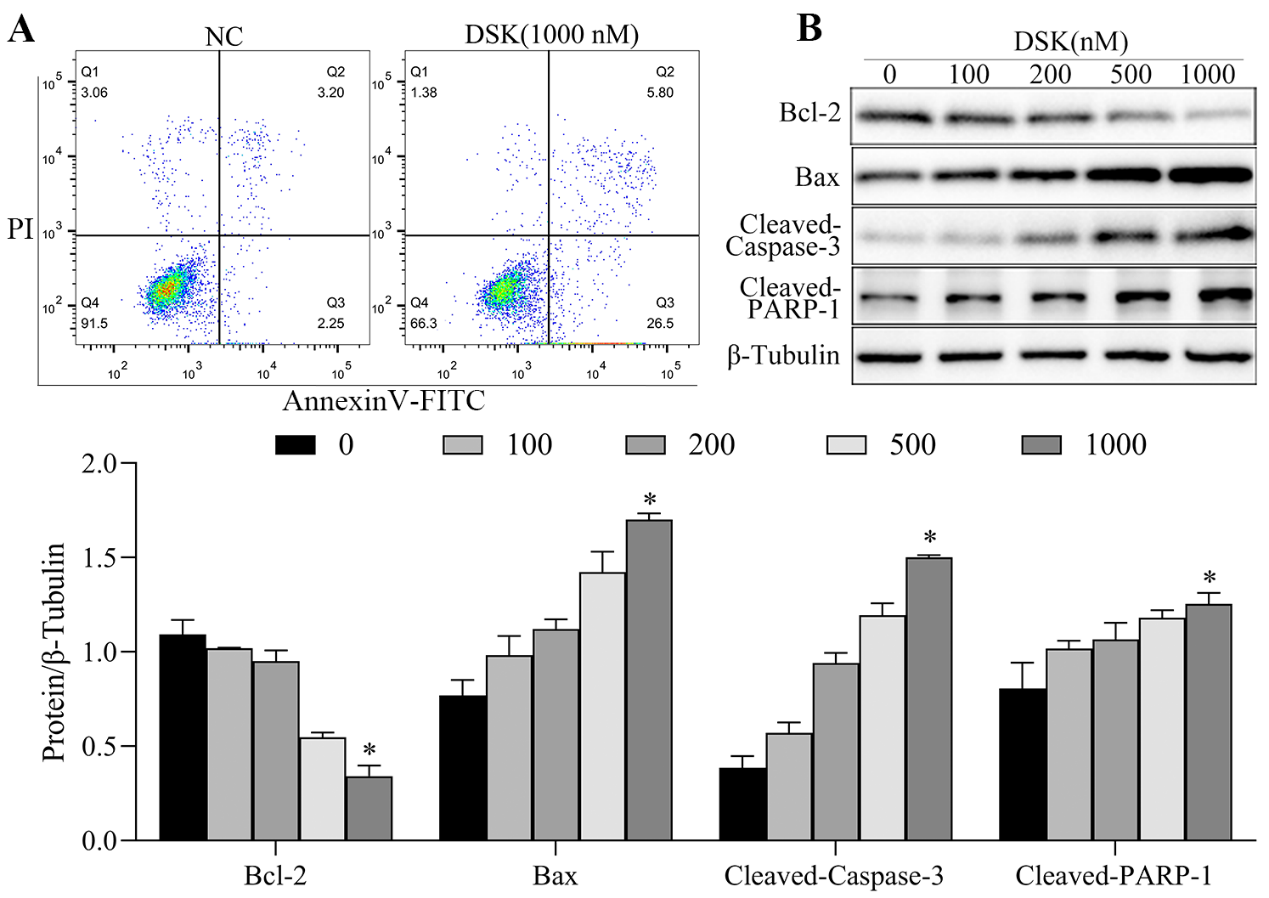
**

**Fig. 2.** DSK induced the apoptosis of HSFs. HSFs were exposed to DSK for 24 h, and the evaluation of the apoptotic ratio was performed using flow cytometry(A). The effect of DSK on apoptosis-associated proteins was examined by western blot(B). Representative data from three separate experiments are shown. **P* < 0.05.


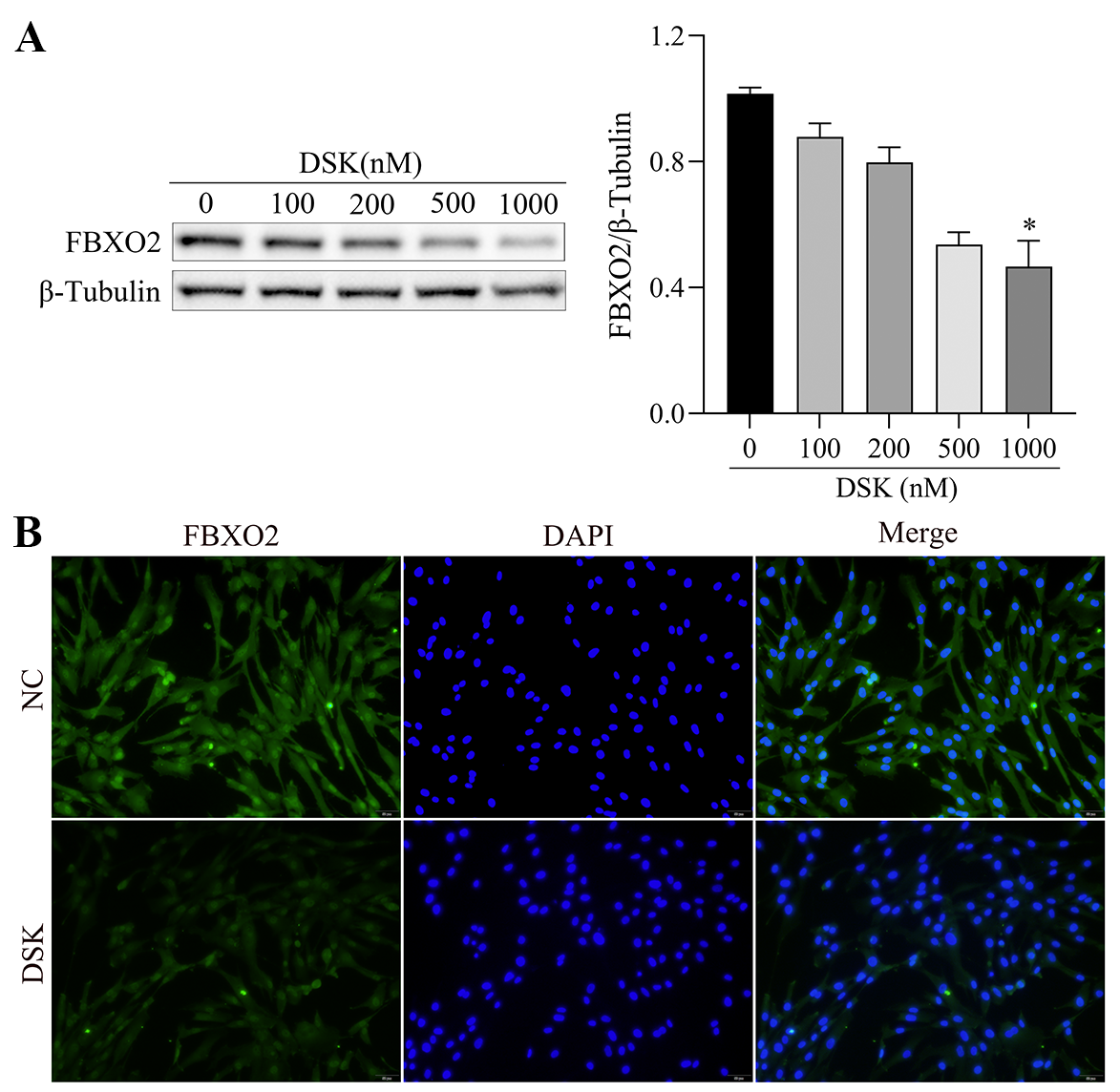


**Fig. 3.** DSK inhibits the expression of FBXO2 in HSFs. HSFs were treated with different concentrations of DSK (0, 100, 200, 500 and 1000nM) and the expressions of FBXO2 were evaluated by western blot (A). The immunofluorescence staining confirmed that DSK decreased FBXO2 expression at a protein level (B). Representative data from three separate experiments are shown. Scale bars: 50 µm **P* < 0.05.


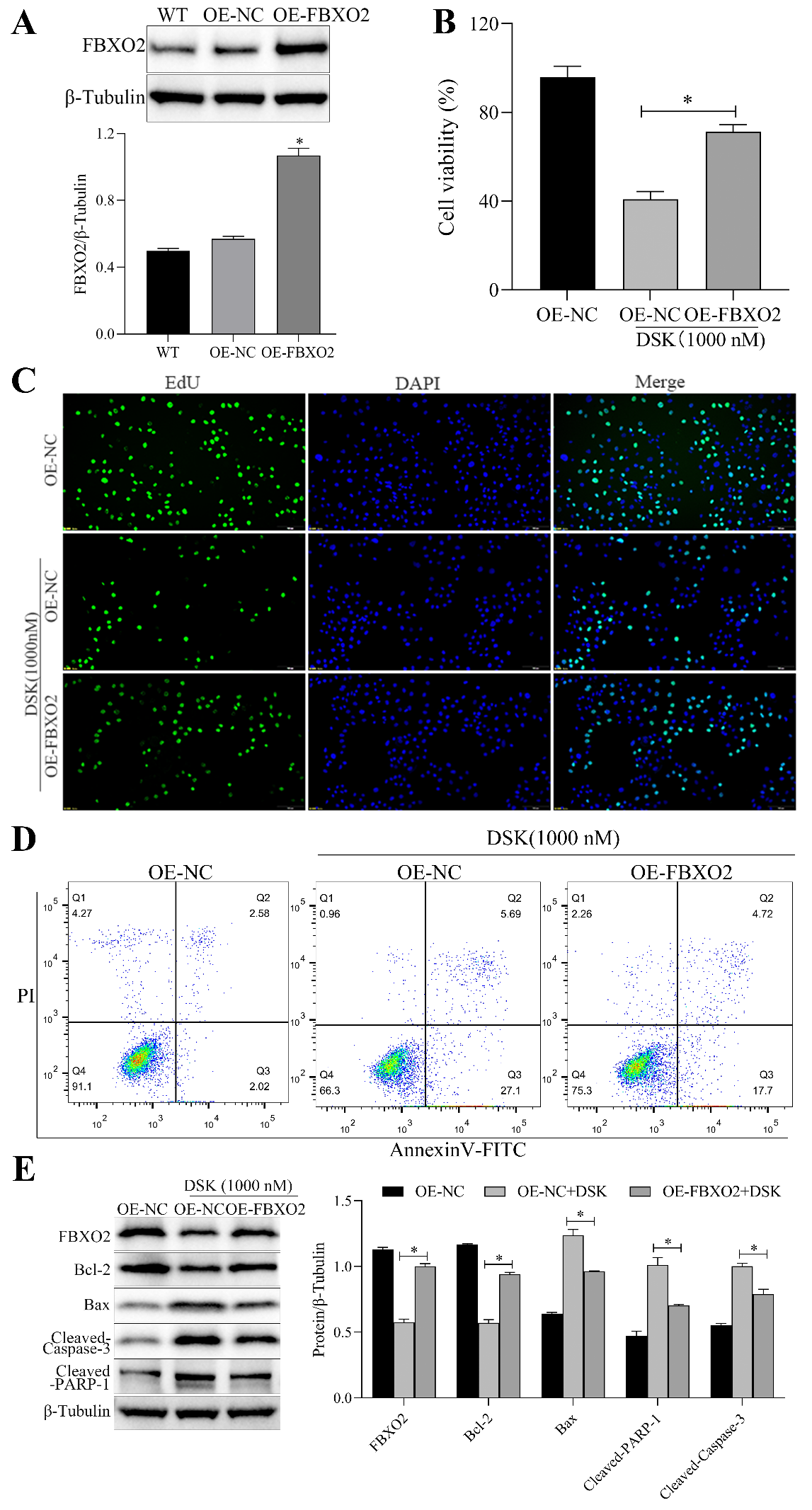


**Fig. 4.** DSK limits the proliferation of HSFs by inhibiting FBXO2 expression. HSFs were infected with oeNC and oeFBXO2 lentivirus before treated with DSK. The transfection efficiency was validated by western blot (A). The cell viability was detected by CCK-8 assay (B). The proliferation of HSFs was detected by EdU assay (C). The cell apoptosis rate was evaluated by flow cytometry analysis(D). The expression of apoptosis-associated proteins was detected by western blot (E). Representative data from three separate experiments are shown. Scale bars: 100 µm **P* < 0.05.


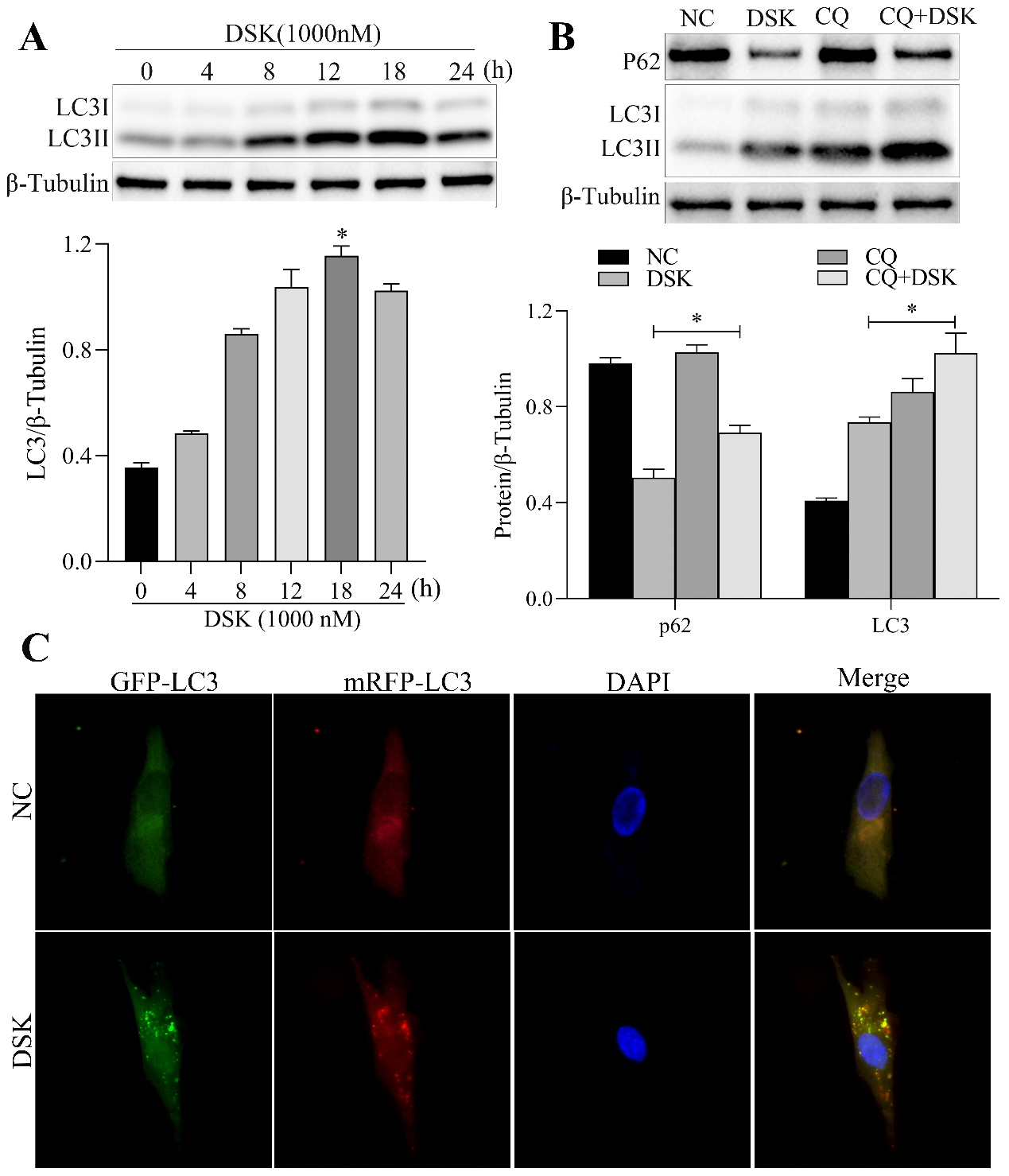


**Fig. 5.** DSK induced autophagy in HSFs. HSFs were treated with 1000nM DSK for different time (0, 4, 8, 12, 18, and 24 h), and the expression of LC3 was detected by western blot (A). HSFs were treated with DSK and/or CQ, and the expressions of LC3 and P62 were evaluated by western blot analysis (B). The formation of autophagosomes was observed by mRFP-GFP-LC3 adenovirus double label assay (C). Representative data from three separate experiments are shown. Scale bars: 50 µm **P* < 0.05.


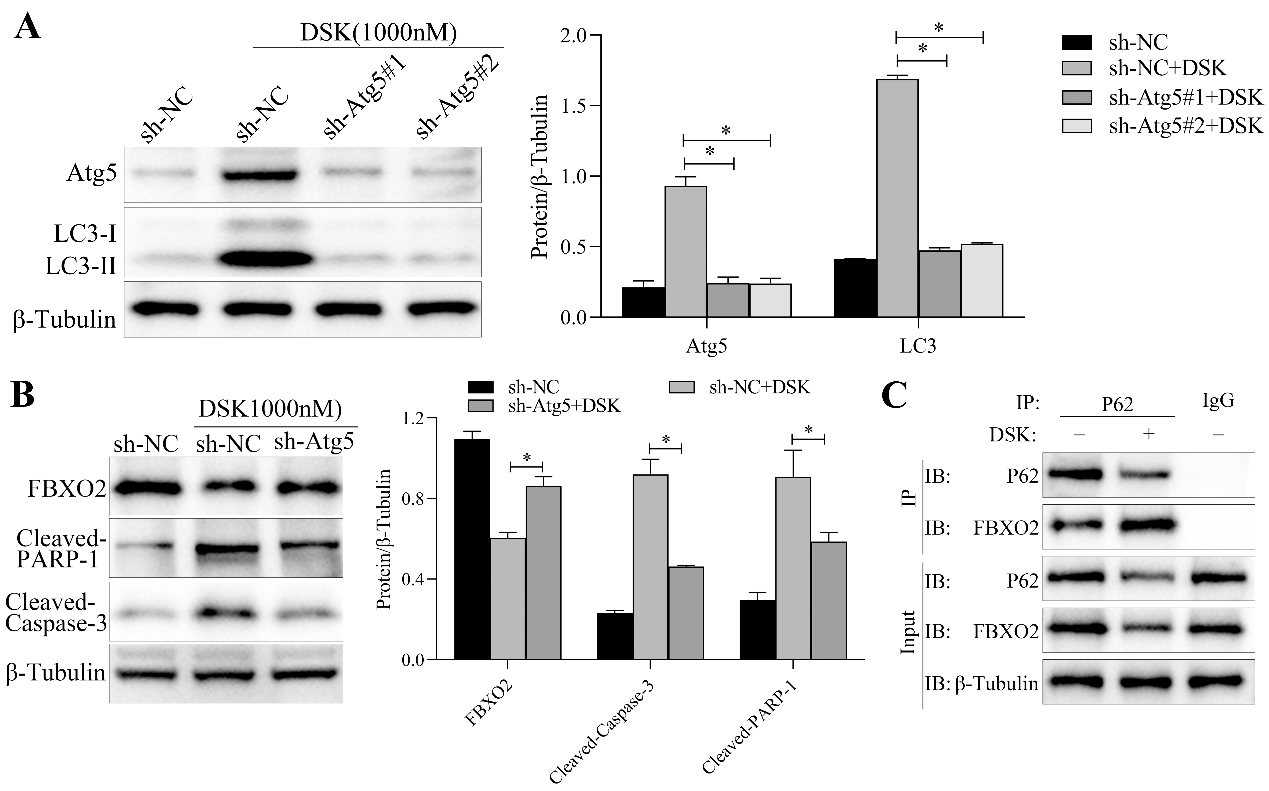


**Fig. 6.** DSK induced autophagy mediates FBXO2 degradation in HSFs. HSFs infected with sh-Atg5 or sh-NC were treated with DSK, and the expression of autophagy-related proteins was detected by western blot (A). The expression of FBXO2 and apoptosis-associated proteins after sh-Atg5 infection were evaluated by western blot (B). The interaction between P62 and FBXO2 was validated by co-immunoprecipitation assay (C). Representative data from three separate experiments are shown. **P* < 0.05.

**
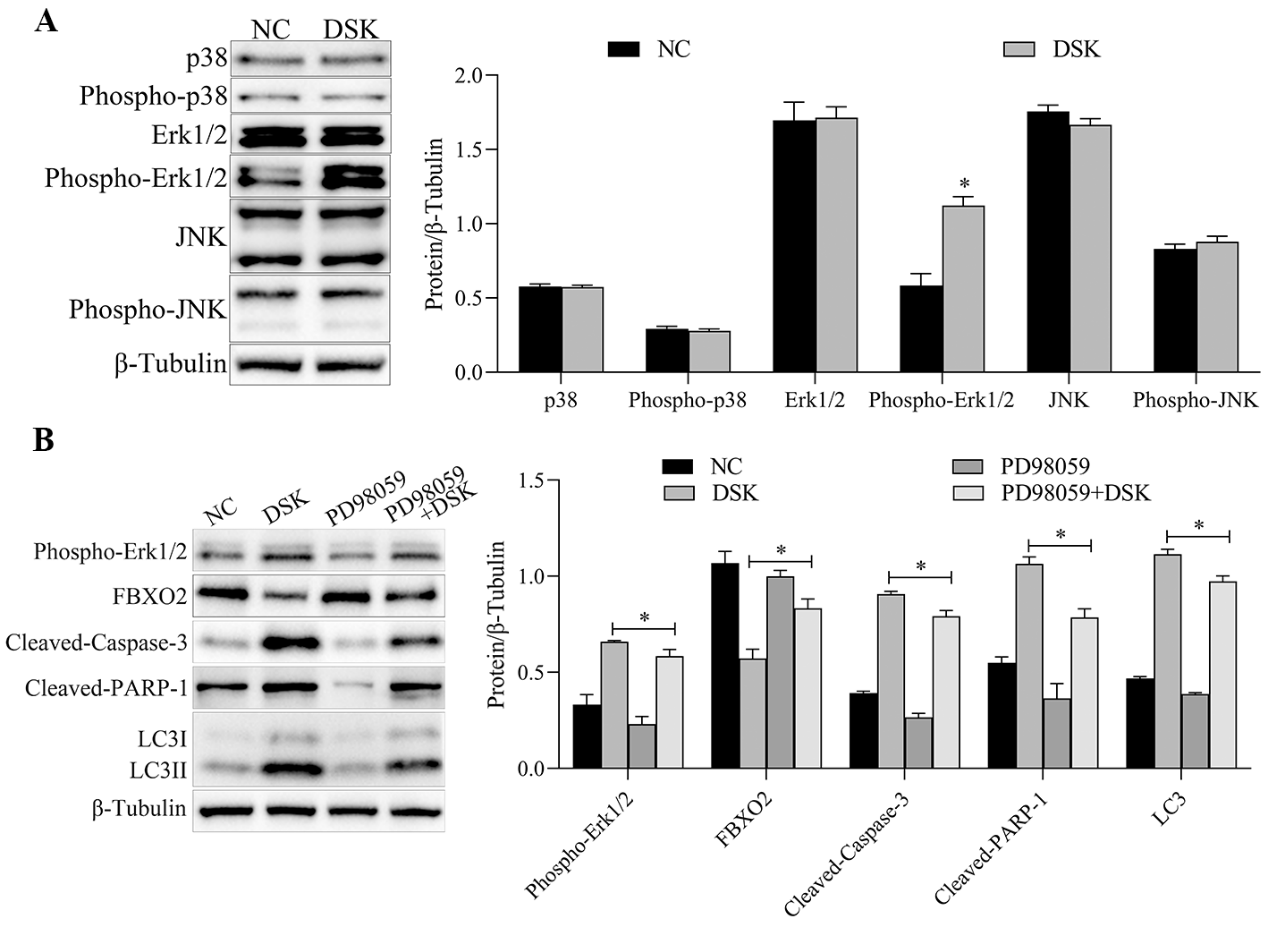
**

**Fig. 7.** DSK induced autophagy and FBXO2 degradation in HSFs by activating ERK1/2 signaling. HSFs were treated with or without DSK for 30min, and the expressions of proteins in MAPK pathway were detected by western blot (A). HSFs were treated with DSK and/or PD98059 for 24 h, followed by western blot analysis to detect the expressions of autophagic markers, apoptosis-associated proteins and FBXO2 (B). Representative data from three separate experiments are shown. **P* < 0.05.
