# Supplementary material for: Deoxyshikonin inhibits growth and induces apoptosis of hypertrophic scar-derived fibroblasts by downregulating FBXO expression through autophagy
Source: Sci Rep. 2026 Apr 28;16:19700. doi: 10.1038/s41598-026-49808-1 (PMC13315721; doi:10.1038/s41598-026-49808-1)

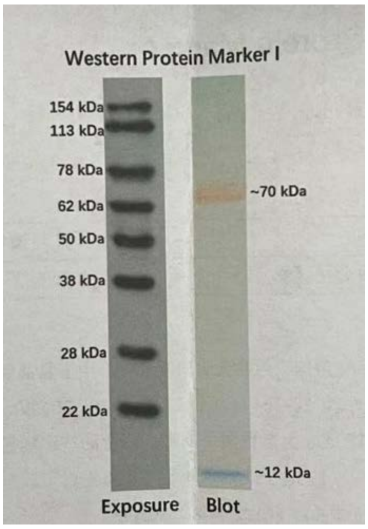

Original Image For Fig 1B

NC

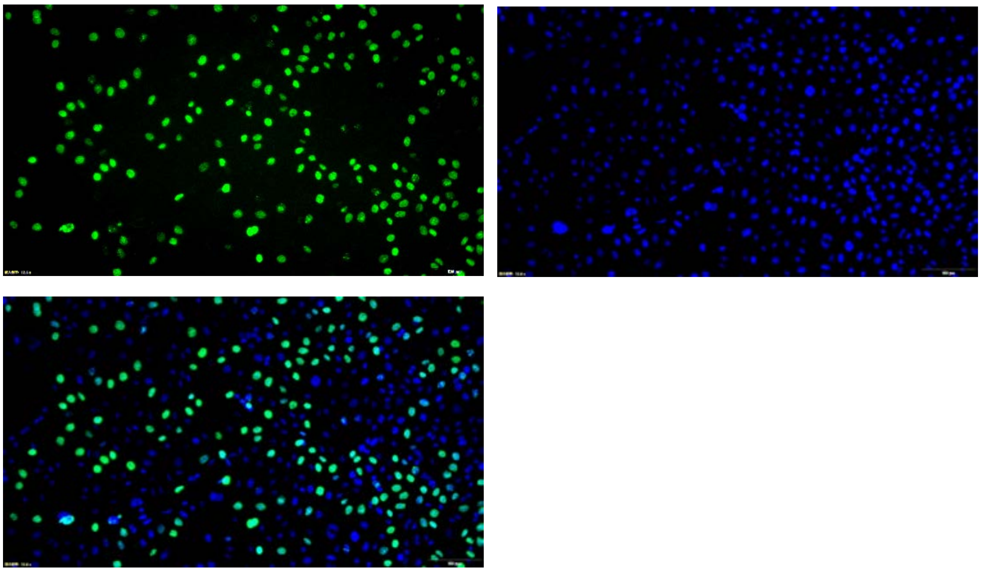

DSK

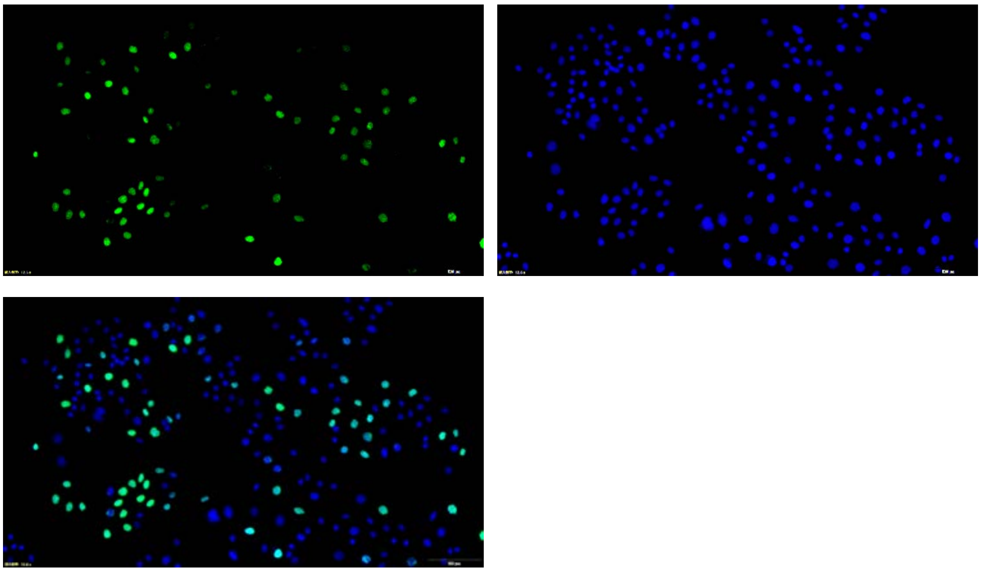

Original Image For Fig 2B

Bcl-2 Bax Cleaved-Caspase-3 Cleaved-PARP-1 β-Tubulin

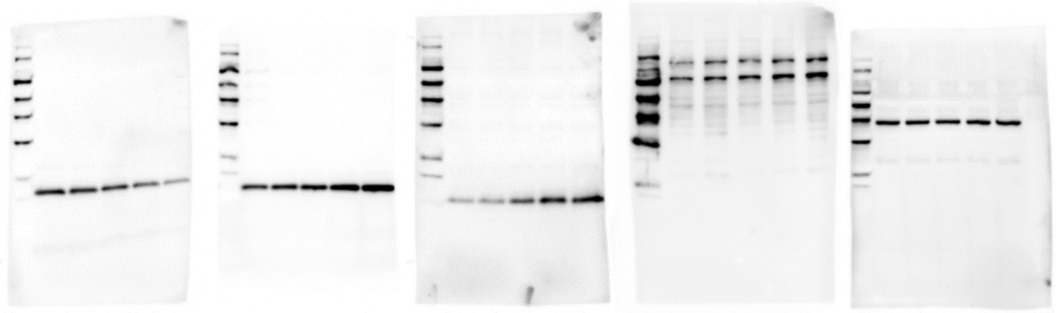

Original Image For Fig 3A

FBXO2 β-Tubulin

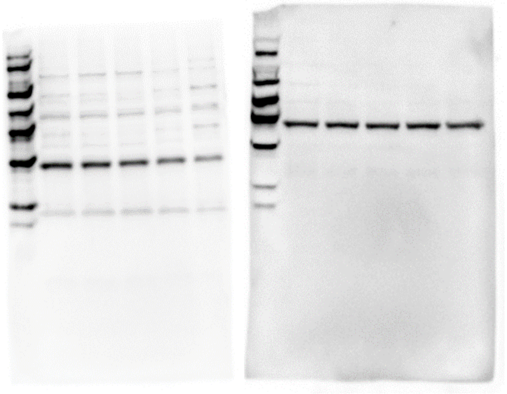

Original Image For Fig 3B

NC

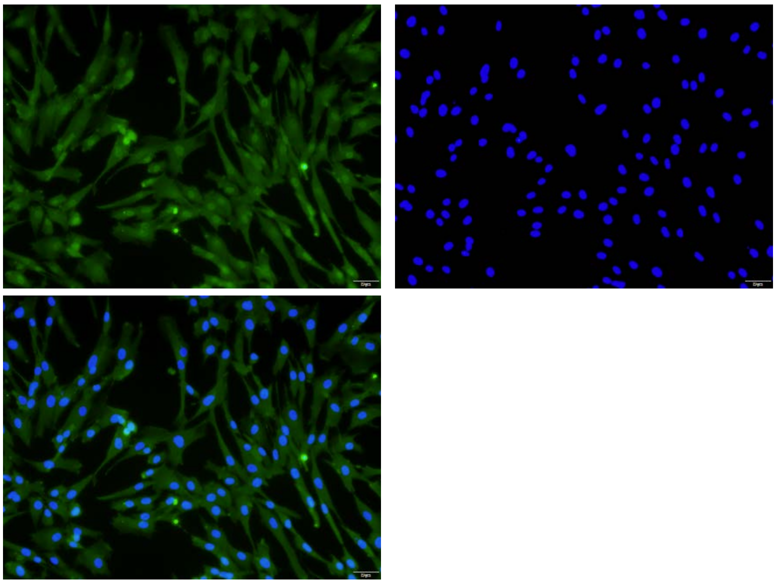

DSK

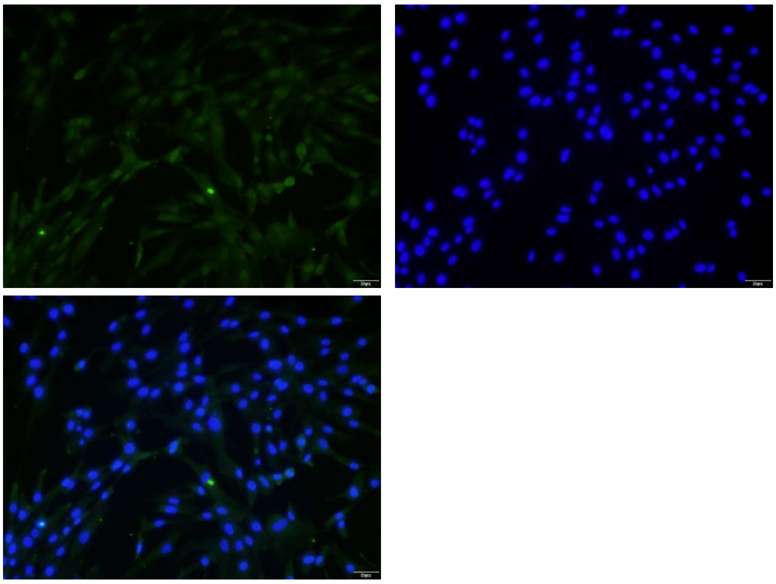

Original Image For Fig 4A

FBXO2 β-Tubulin

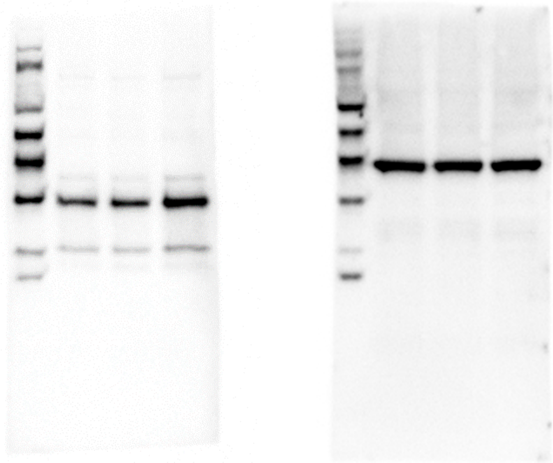

Original Image For Fig 4C

OE-NC

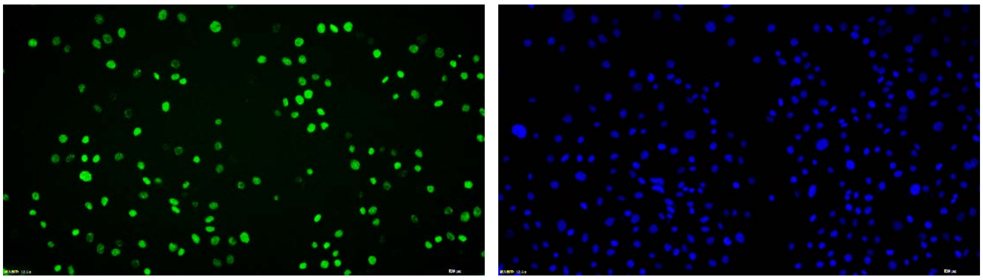

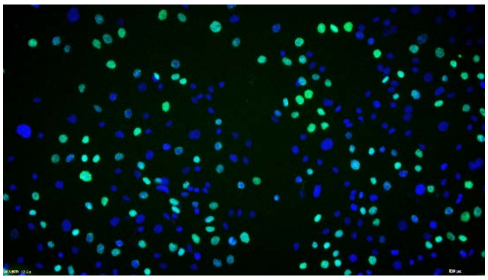

OE-NC+DSK

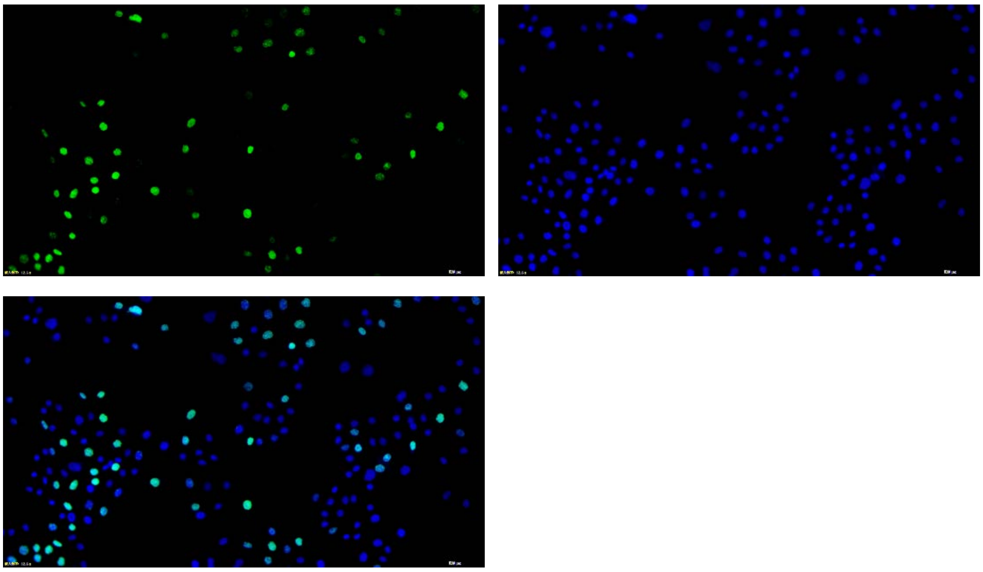

OE-FBXO2+DSK

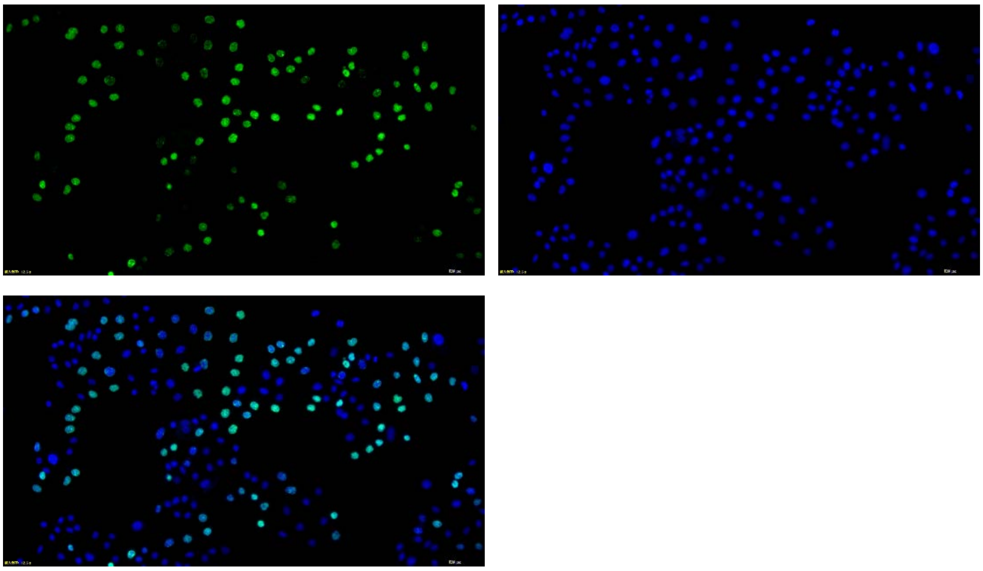

Original Image For Fig 4E

FBXO2 Bcl-2 Bax Cleaved-Caspase-3 Cleaved-PARP-1 β-Tubulin

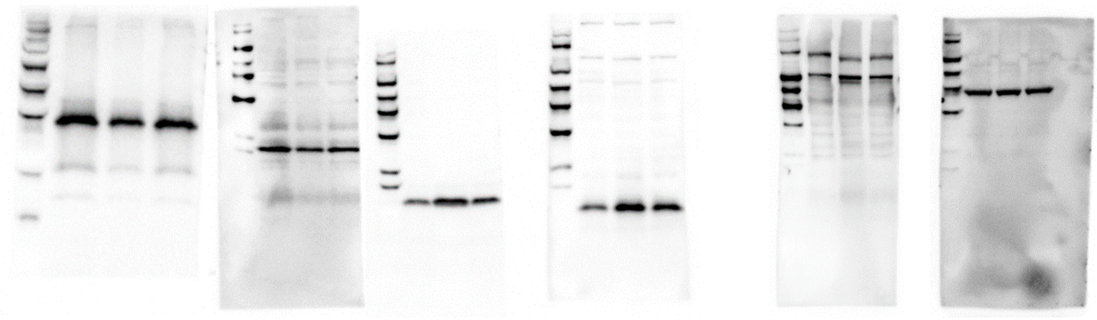

Original Image For Fig 5A

LC3 β-Tubulin

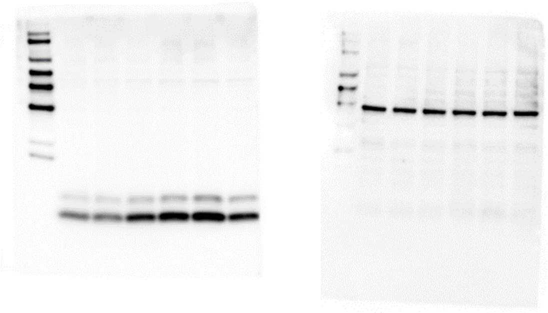

Original Image For Fig 5B

P62 LC3 β-Tubulin

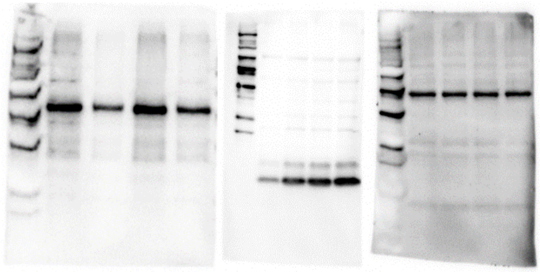

Original Image For Fig 5C

NC

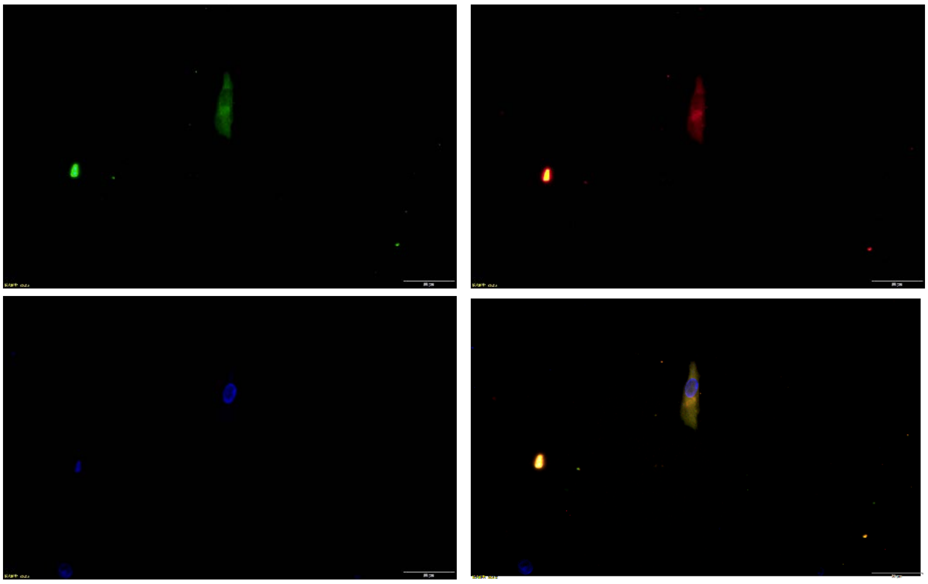

DSK

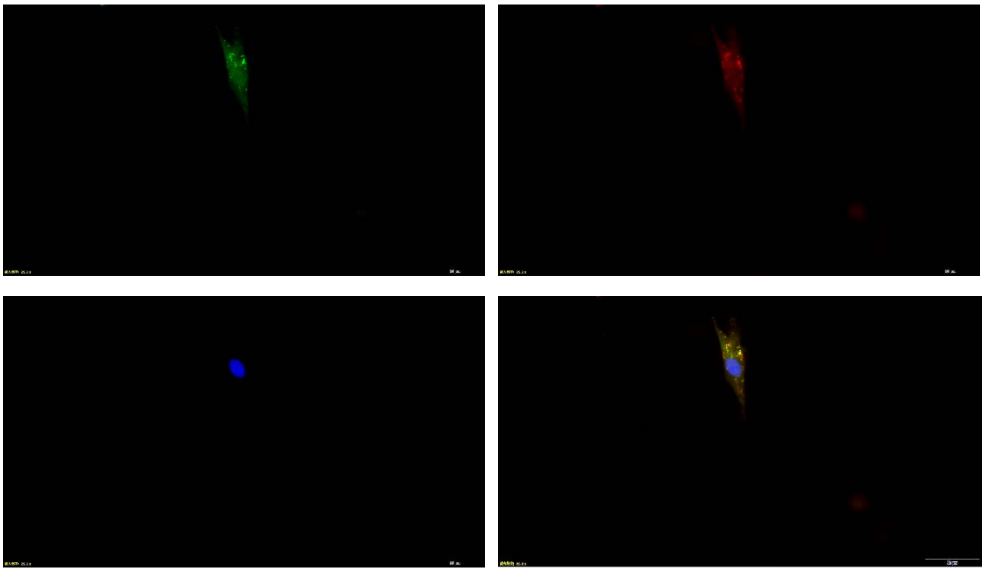

Original Image For Fig 6A

Atg5 LC3 β-Tubulin

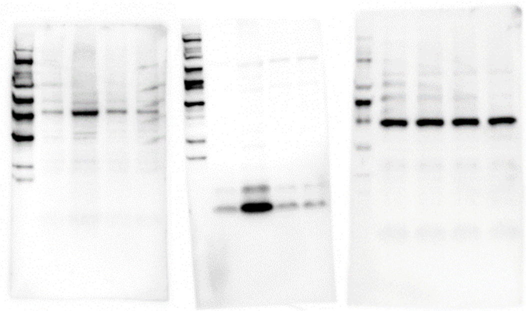

Original Image For Fig 6B

FBXO2 Cleaved-PARP-1 Cleaved-Caspase-3 β-Tubulin

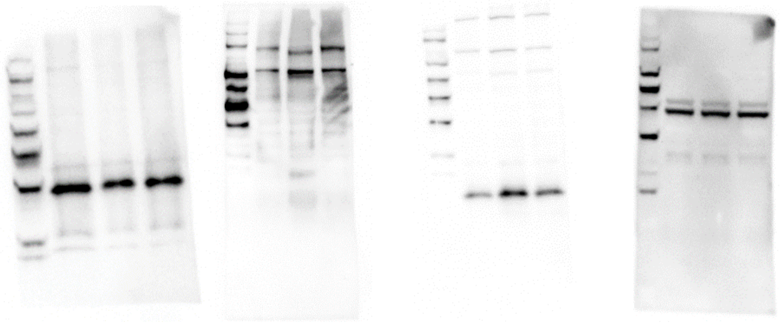

Original Image For Fig 6C

P62 FBXO2 P62 FBXO2 β-Tubulin

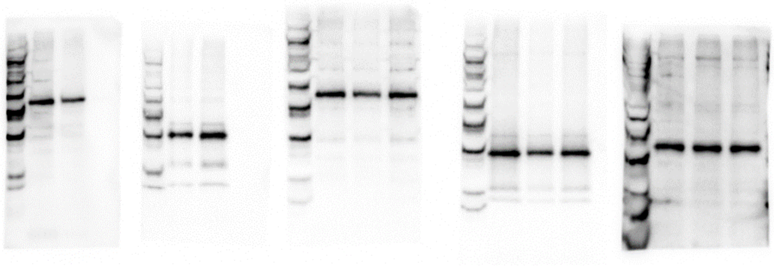

Original Image For Fig 7A

P38 p-p38 Erk1/2 p-Erk1/2 JNK P-JNK β-Tubulin

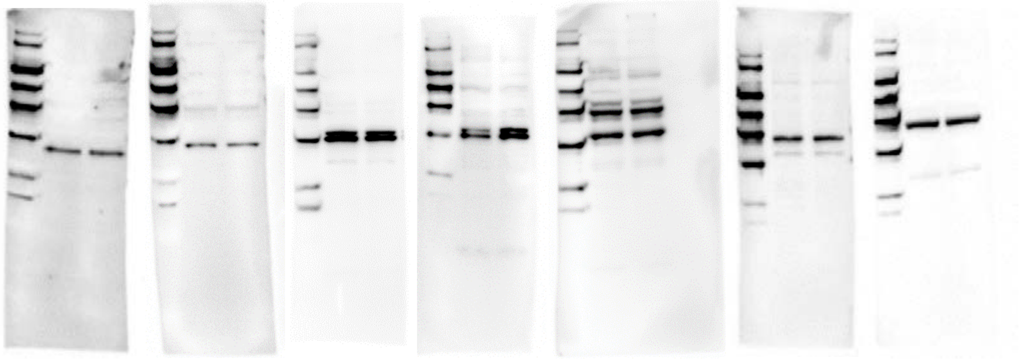

Original Image For Fig 7B

p-Erk1/2 FBXO2 Cleaved-Caspase-3 Cleaved-PARP-1 LC3 β-Tubulin

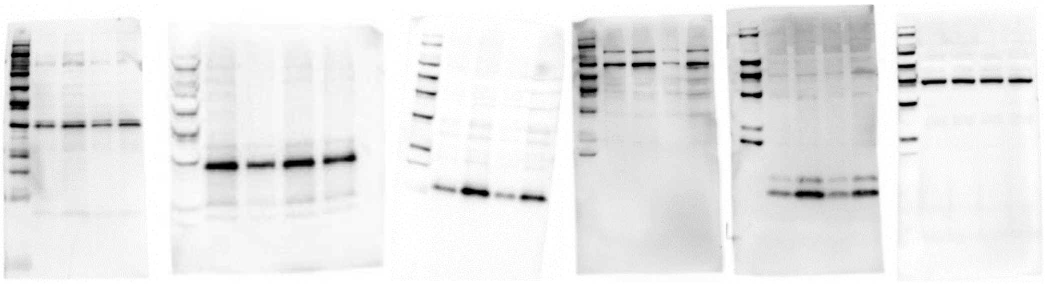

Supplement: Supplementary file 2 — Supplementary Material 2 [file 41598_2026_49808_MOESM2_ESM.pdf]
