# Supplementary figures and images for: Deoxyshikonin inhibits growth and induces apoptosis of hypertrophic scar-derived fibroblasts by downregulating FBXO expression through autophagy
Source: Sci Rep. 2026 Apr 28;16:19700. doi: 10.1038/s41598-026-49808-1 (PMC13315721; doi:10.1038/s41598-026-49808-1)

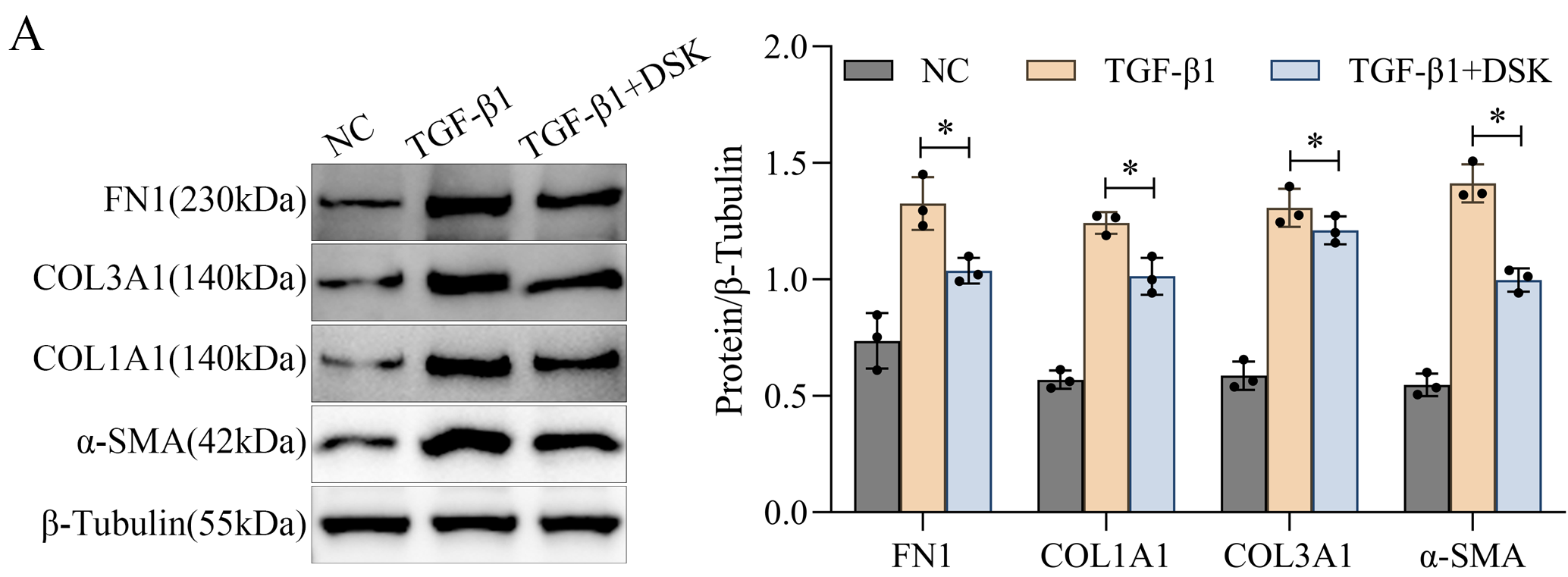

Supplement: Supplementary file 3 — Supplementary Material 3 [file 41598_2026_49808_MOESM3_ESM.tif]

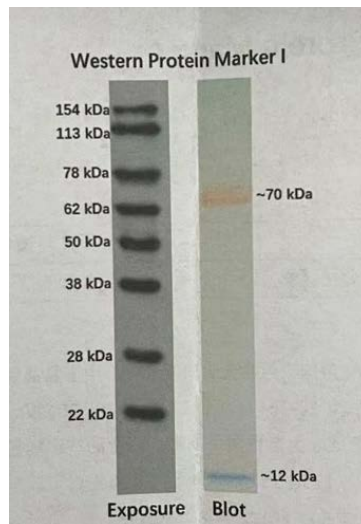

Original Image For S1

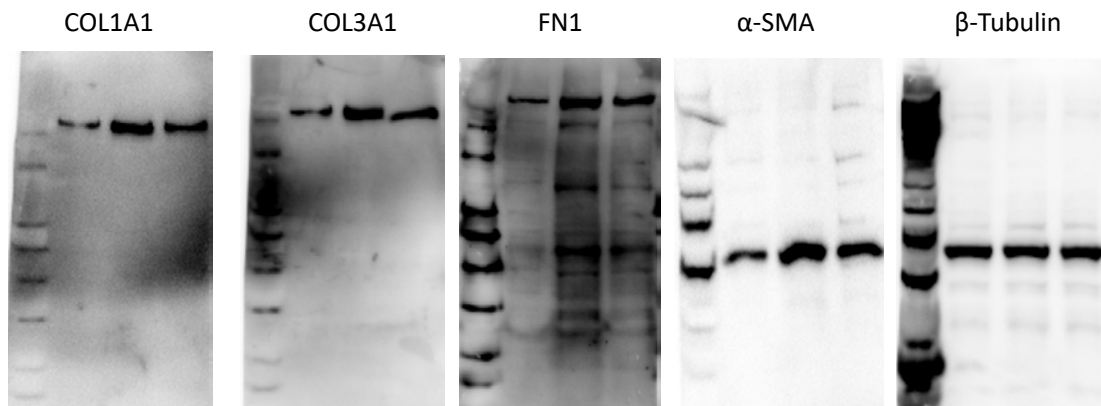

Supplement: Supplementary file 4 — Supplementary Material 4 [file 41598_2026_49808_MOESM4_ESM.pdf]
